# Supplementary material for: GPR50 Promotes Hepatocellular Carcinoma Progression via the Notch Signaling Pathway through Direct Interaction with ADAM17
Source: Mol Ther Oncolytics. 2020 Apr 14;17:332–49. doi: 10.1016/j.omto.2020.04.002 (PMC7210388; doi:10.1016/j.omto.2020.04.002)
Supplement: Document S1. Figures S1 and S2 and Tables S1–S3 [file mmc1.pdf]

OMTO, Volume *17*

Supplemental Information

GPR50 Promotes Hepatocellular Carcinoma Progression via the Notch Signaling Pathway through Direct Interaction with ADAM17

Subbroto Kumar Saha, Hye Yeon Choi, Gwang-Mo Yang, Polash Kumar Biswas, Kyeongseok Kim, Geun-Ho Kang, Minchan Gil, and Ssang-Goo Cho

Supplemental Information:

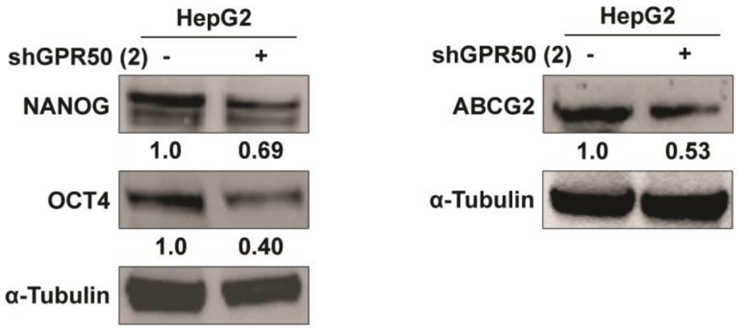

Supplementary Figure 1. Knockdown of *GPR50* led to suppressed protein expression of stemness and drug resistant markers in HCC. Protein expression of stemness markers (NANOG and OCT4) and drug resistant marker (ABCG2) were analyzed by western blot. α-Tubulin was used as an internal standard.

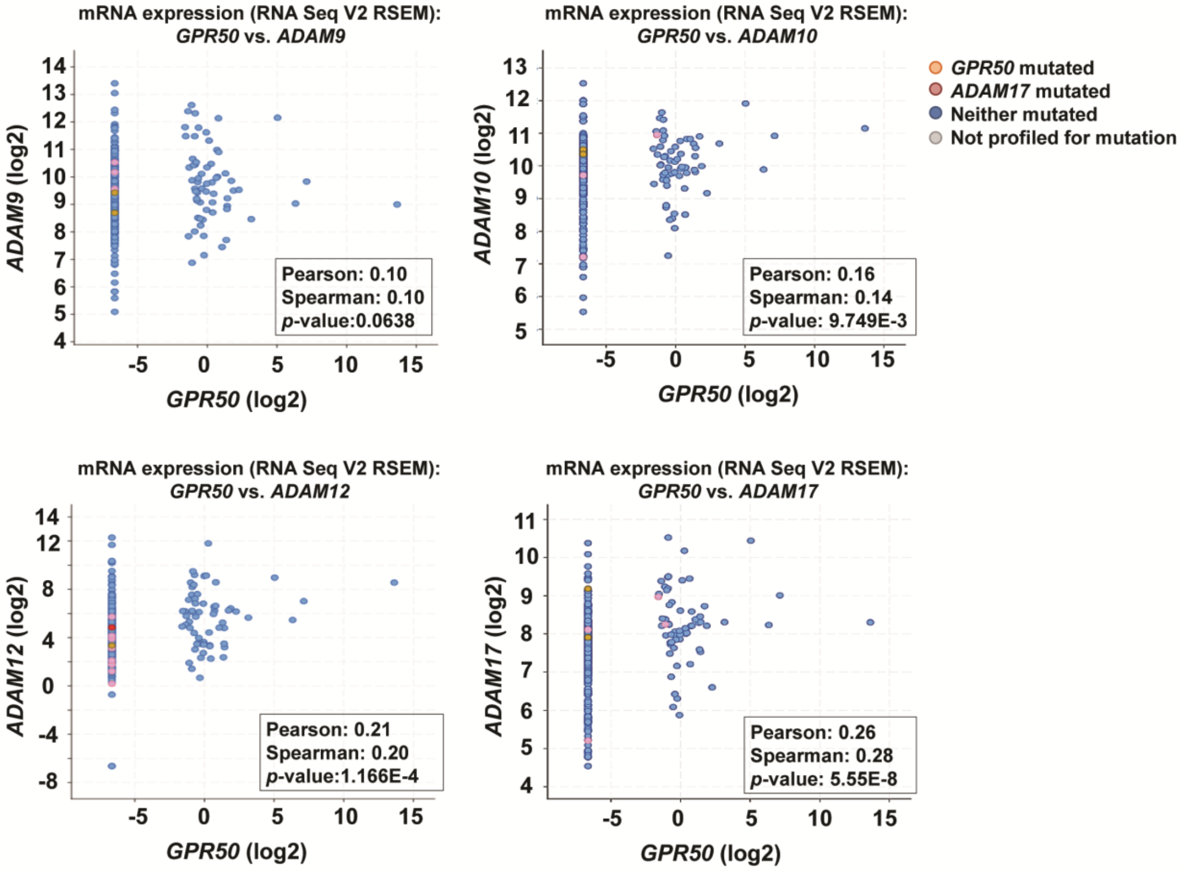

Supplementary Figure 2. GPR50 is positively co-expressed with ADAM family genes including *ADAM9*, *ADAM10*, *ADAM12*, and *ADAM17* in HCC. The co-expression data was plotted using TCGA data and downloaded from cBioPortal web.

Supplementary Table 1. *GPR50* is differentially expressed in various cancer types. Analysis of GEO datasets for *GPR50* mRNA expression in BRC, CEC, ESC, HCC, and LUC compared with normal breast, cervical, esophageal, liver, and lung tissue.

GEO/EMBL-EBI accession no.	Median expression (Normal sample)	Median expression (Cancer sample)	*p*-value
**Breast**			
GSE8977	-0.129 (15)	-0.459 (7)	0.019
EGAS00000000083	-0.14 (144)	-0.17 (14)	0.039
**Cervical**			
GSE7410	0.194 (5)	-0.131 (40)	1.75E-4
GSE9750	-2.66 (21)	-2.983 (32)	0.132
**Esophagus**			
GSE13898	0.225 (28)	0.224 (75)	7.29E-6
GSE23400	-1.199 (53)	-1.296 (53)	0.003
**Liver**			
GSE14520	3.601 (220)	3.524 (225)	<0.0001
GSE54236	4.19 (80)	4.323 (78)	0.475
**Lung**			
GSE31210	-4.885 (20)	-4.352 (226)	0.003
GSE2514	-1.724 (19)	-1.617 (20)	0.071

Supplementary Table 2. List of primers used for knockdown and overexpression of GPR50.

**Accession no.**	**shRNA**	**Oligomers (5′–3′)**	**Target site**
NM_004224.3	*GPR50*-(1)	TCGTGGGTTTCTGCTACGTGAG	747-767
	GPR50-(2)	GCTGACTCTGTCCATTTCAAG	1480-1500
	Scrambled	CCTAAGGTTAAGTCGCCCTCGCTC	Non-specific
**Plasmid name**	**Gene**	**Forward (5′–3′)**	**Reverse (5′–3′)**
pGEMR-T easy vector	*GPR50*	TATtctagaAGAGAGGGAGGCACGCTT (*Xba*I)	TATggatccTGCAGTAAGGCATCTCAC (*BamH*I)
pCDH-EF1-MCS-T2A-copGFP	*GPR50*		CGCggatccCACAGCCATTTCATCAGG (*BamH*I)

Underlined sequences represent the restriction sites.

Supplementary Table 3. Primer sequences used for qRT-PCR.

Accession no.	Gene	Froward Primer (5'→3')	Reverse Primer (5'→3')
NM_004224.3	*GPR50*	CCATCGTTGTAGACCTAATCG	CAGCATCAAAGGGTATGGGTA
NM_002046.5	*GAPDH*	AATCCCATCACCATCTTCCAG	CACGATACCAAAGTTGTCATGG
NM_001285986.1	*OCT4*	GTCCCAGGACATCAAAGCTC	CTCCAGGTTGCCTCTCACTC
NM_003106.3	*SOX2*	ACACCAATCCCATCCACACT	GCAAGAAGCCTCTCCTTGAA
NM_024865.3	*NANOG*	ATACCTCAGCCTCCAGCAGA	GCAGGACTGCAGAGATTCCT
NM_002467.4	*c-MYC*	CTCGGATTCTCTGCTCTC	TCGCCTCTTGACATTCTC
NM_001314052.1	*KLF4*	GAACTGACCAGGCACTACCG	TTCTGGCAGTGTGGGTCATA
NM_001348946.1	*P-GP*	GAGGAAGACATGACCAGGTA	CTGTCGCATTATAGCATGAA
NM_004827.2	*ABCG2*	TTATCCGTGGTGTGTCTGGAG	TCCTGCTTGGAAGGCTCTATG
NM_004996.3	*ABCC1*	GCCGGTGAAGGTTGTGTACT	CTGACGAAGCAGATGTGGAA
NM_001163993.2	*ABCB5*	GAGAGACAGTCGCCTTGGTC	CCACGATTGTAGTCCGACCT
NM_000689.4	*ALDH1A1*	CTGCTGGCGACAATGGAGT	GTCAGCCCAACCTGCACAG
NM_000214.3	*JAG1*	CGGGATTTGGTTAATGGTTATC	ATAGTCACTGGCACGGTTGTAGCAC
NM_002226.5	*JAG2*	GGTCGTACTTGCACTCACAATACC	GTAGCAAGGCAGAGGGTTGC
NM_005618.4	*DLL1*	CCAAGCCCTGCAAGAATGGA	GGTGGGCAGGTACAGGAGTA
NM_016941.3	*DLL3*	GAGACACCCAGGTCCTTTGA	CAGTGGCAGATGTAGGCAGA
NM_019074.4	*DLL4*	GTGGGTCAGAACTGGTTATGGA	TGCAGATGACCCGGTAAGAGT
NM_017617.3	*NOTCH1*	GACGGACCCAACACTTACAC	TCAGGCAGAAGCAGAGGTAG
NM_014757.4	*MAML1*	CACCAGCCACCGAGTAACTT	AACAGGGAGTTCTGCTCGTG
NM_005349.3	*RBPjK*	GAACAAATGGAACGCGATGG	GATGACTTTTATCCGCTTGCTG
NM_005524.3	*HES1*	GGCTAAGGTGTTTGGAGGCT	GGTGGGTTGGGGAGTTTAGG
NM_001130145.3	*YAP1*	TGAACAAACGTCCAGCAAGATAC	CAGCCCCCAAAATGAACAGTAG
NM_012242.4	*DKK1*	GGGCGGGAATAAGTACCAG	CATAGCGTGACGCATGCAG
NM_005269.3	*GLI1*	TTCCTACCAGAGTCCCAAGT	CCCTATGTGAAGCCCTATTT
NM_003816.3	*ADAM9*	GGTGCTGGTGATGTGCTG	CTGCGTGGCTCCTTGAAC
NM_001110.4	*ADAM10*	ATGGATTGTGGCTCATTGGT	TGCCTGGAAGTGGTTTAGGA
NM_001288975.1	*ADAM12*	CAGGAAGGACTTGGAGAC	AGCAGCGATTCATACATTC
NM_003183.6	*ADAM17*	GTATCTGAACAACGACACCTG	CCTCCTGGCACTTCTTCTG
NM_138473.3	*SP1*	TCCAGACCATTAACCTCAGTGC	TGTATTCCATCACCACCAGCC
NM_003110.6	*SP2*	CCAGCCTACCCCAAGGAAAC	GGGAGCCCTGAATCTGAAGTAT
NM_001172712.1	*SP3*	GCTTGCACCTGTCCCAACTGTA	CTCCAGAATGCCAACGCAGA
NM_003112.5	*SP4*	ATGGCTACAGAAGGAGGGAAAAC	TTGACCAGGGGTGGAAGAATTAC
