# Supplementary material for: GPR50 Promotes Hepatocellular Carcinoma Progression via the Notch Signaling Pathway through Direct Interaction with ADAM17
Source: Mol Ther Oncolytics. 2020 Apr 14;17:332–49. doi: 10.1016/j.omto.2020.04.002 (PMC7210388; doi:10.1016/j.omto.2020.04.002)
Supplement: Document S2. Article plus Supplemental Information [file mmc2.pdf]

Molecular Therapy
Oncolytics
Original Article

AMERICAN SOCIETY of GENE & CELL THERAPY

GPR50 Promotes Hepatocellular Carcinoma Progression via the Notch Signaling Pathway through Direct Interaction with ADAM17

Subbroto Kumar Saha,[1] Hye Yeon Choi,[1] Gwang-Mo Yang,[1] Polash Kumar Biswas,[1] Kyeongseok Kim,[1] Geun-Ho Kang,[1] Minchan Gil,[1] and Ssang-Goo Cho[1]

[1]Department of Stem Cell and Regenerative Biotechnology, Konkuk University, Seoul 05029, Republic of Korea

Hepatocellular carcinoma (HCC) is a leading cause of cancer-related death worldwide, and it is thus critical to identify novel molecular biomarkers of HCC prognosis and elucidate the molecular mechanisms underlying HCC progression. Here, we show that G-protein-coupled receptor 50 (*GPR50*) in HCC is overexpressed and that *GPR50* knockdown may downregulate cancer cell progression through attenuation of the Notch signaling pathway. *GPR50* knockdown was found to reduce HCC progression by inactivating Notch signaling in a ligand-independent manner through a disintegrin and metalloproteinase metallopeptidase domain 17 (ADAM17), a proteolytic enzyme that cleaves the Notch receptor, which was corroborated by *GPR50* overexpression in hepatocytes. *GPR50* silencing also downregulated transcription and translation of ADAM17 through the AKT/specificity protein-1 (SP1) signaling axis. Notably, GPR50 was found to directly interact with ADAM17. Overall, we demonstrate a novel GPR50-mediated regulation of the ADAM17-Notch signaling pathway, which can provide insights into HCC progression and prognosis and development of Notch-based HCC treatment strategies.

INTRODUCTION

Hepatocellular carcinoma (HCC) is the fourth-leading cause of cancer-related death worldwide.[1] Despite significant progress, more than 70% of HCC patients cannot be diagnosed at an early stage, which is easier to treat via local ablation, hepatic resection, or liver transplantation.[2,3] Treatment is more difficult during later stages, and cancer recurrence and metastatic rates increase, leading to increased mortality.[4–6] Therefore, the identification of novel molecular biomarkers of HCC and its prognosis and the elucidation of the detailed molecular mechanisms underlying HCC progression are of great importance.

G-protein-coupled receptors (GPCRs), or seven transmembrane (7TM)-spanning proteins, represent the largest class of cell surface receptor proteins, with approximately 800 members. GPCRs are important proteins in drug development and are reported to be key targets for more than 30% of drugs on the market.[7] GPCRs were found to play dynamic roles in cancer development and progression, including survival and tumor growth.[8,9] Moreover, around 100 GPCRs have been classified as orphan GPCRs, because their endogenous ligands remain unidentified; however, several of them were reported to function in a ligand-independent manner.[10,11] They can facilitate signal transduction from the extracellular environment to intracellular effectors[12] and mediate physiological and disease progression.[13] A recent endeavor has strengthened the need to explore the vital role of GPCRs and their ligands, such as chemokines, lysophosphatidic acid (LPA), serine proteases (PAR1), and sphingosine 1-phosphate (S1P), in metastasis.[14–16] Previously, pro-metastatic functions have been ascribed to numerous orphan GPCRs, such as GPR64, GPR116, and GPR161,[10,17,18] highlighting the value of studying these receptors as novel therapeutic targets for preventing cancer metastasis.

GPR50, another member of orphan GPCRs, exhibits high sequence similarity with the melatonin receptors MT1 and MT2; however, melatonin is not a GPR50 ligand.[19,20] GPR50 was reported to be associated with bipolar-affective disorder, lipid metabolism, thermogenesis, adipogenesis, and neuronal development.[21–25] In our previous study, several GPCRs, including GPR50, were claimed to be involved in the reprogramming of somatic cells to cancer stem cells and in the maintenance of stemness function.[26] Recent studies also reported that *GPR50* can act as a tumor suppressor in breast cancer (BRC);[27,28] however, there is limited research on the role of *GPR50* in cancer progression.

In this study, we aimed to uncover the role of *GPR50* in HCC progression and prognosis. As *GPR50* was described as a tumor suppressor in breast cancer, we examined whether *GPR50* plays an oncogenic or a tumor-suppressor role in HCC. We found that *GPR50* is overexpressed in HCC and that *GPR50* knockdown can suppress HCC progression by downregulating the Notch signaling pathway. Our

Received 16 March 2020; accepted 6 April 2020;
https://doi.org/10.1016/j.omto.2020.04.002.

Correspondence: Ssang-goo Cho, Department of Stem Cell and Regenerative Biotechnology, Konkuk University, 120 Neungdong-ro, Gwangjin-gu, Seoul 05029, Republic of Korea.
E-mail: ssangoo@konkuk.ac.kr

 © 2020 The Author(s).
This is an open access article under the CC BY license (http://creativecommons.org/licenses/by/4.0/).

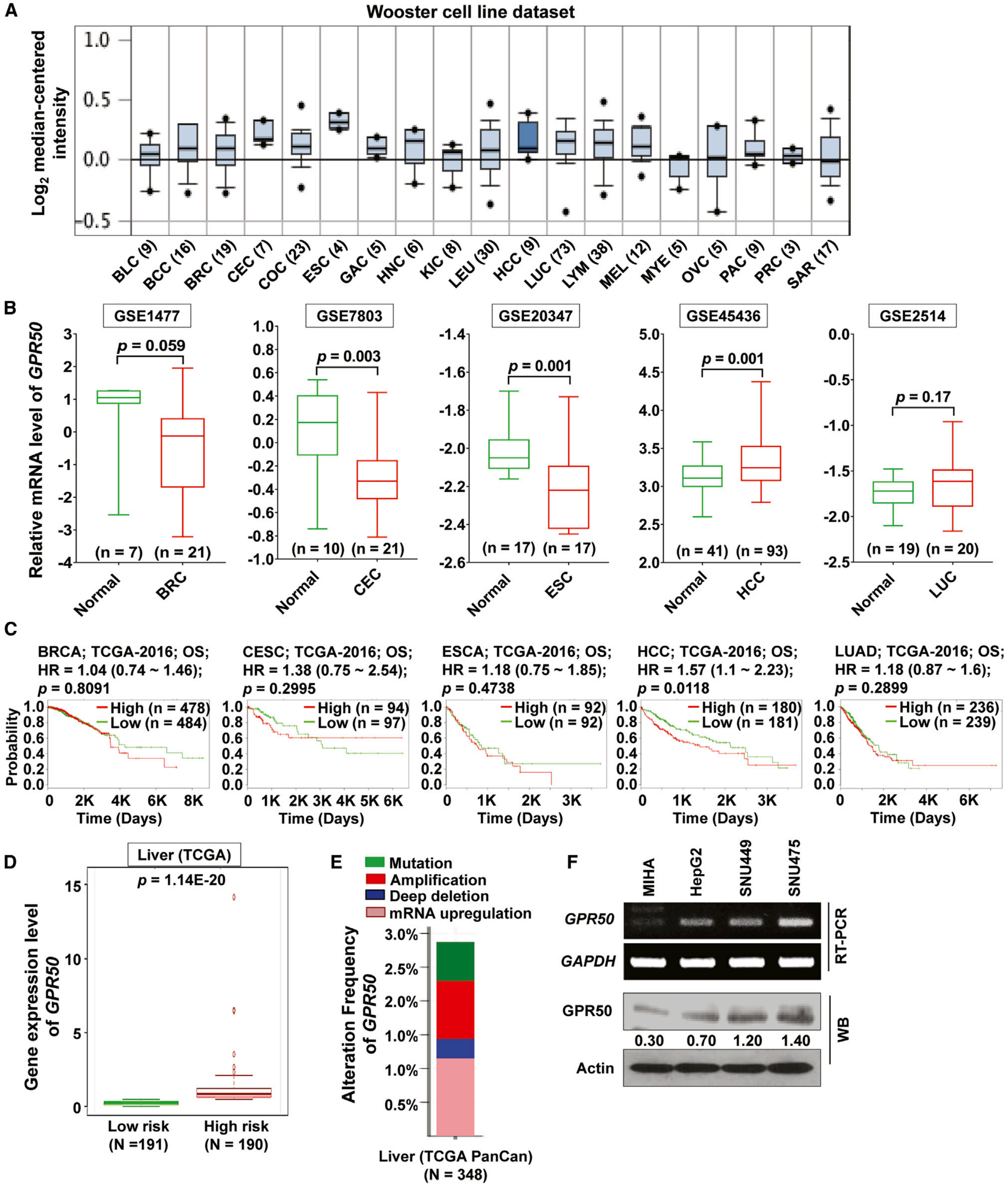

Figure 1. *GPR50* Is Differentially Expressed in Various Cancer Types

(A) Oncomine database Log2 median-centered expression intensities for *GPR50* genes in various cancers, such as bladder (BLC; n = 9), brain and CNS cancer (BCC; n = 16), breast (BRC; n = 19), cervical (CEC; n = 7), colorectal (COC; n = 23), esophageal (ESC; n = 4), gastric (GAC; n = 5), head and neck (HNC; n = 6), kidney (KIC; n = 8), leukemia

(legend continued on next page)

findings also indicate that GPR50 forms a novel molecular complex with a disintegrin and metalloproteinase (ADAM) metallopeptidase domain 17 (ADAM17) and regulates ADAM17 activity, activating the Notch signaling pathway in HCC in a ligand-independent manner. This pathway is also partially regulated by GPR50-mediated *ADAM17* transcription via the noncanonical AKT/specificity protein 1 (SP1) axis. Thus, our results support the potential of targeting HCC via the GPR50/ADAM17/Notch signaling pathway.

RESULTS

GPR50 Is Differentially Expressed in Various Cancers and Associated with Liver Cancer Prognosis

Using the Oncomine database (https://www.oncomine.org/resource/login.html) to examine the expression status of *GPR50* in various cancers, we found dysregulated *GPR50* expression (Wooster cell line dataset) that was especially enhanced in BRC, cervical (CEC), esophagus (ESC), liver (HCC), and lung (LUC) cancers (Figure 1A). Subsequently, we analyzed *GPR50* mRNA expression in these cancers using several Gene Expression Omnibus (GEO) datasets. The GEO data showed that *GPR50* expression was significantly upregulated in liver cancers (i.e., HCC) and downregulated in breast, cervical, esophagus, and lung cancers (Figure 1B; Table S1), which is in contrast with the expression patterns in the Oncomine database. Moreover, we analyzed the association between prognosis and *GPR50* expression in various cancer patients using The Cancer Genome Atlas (TCGA) database via the SurvExpress web. Among the indicated cancers, high *GPR50* expression exhibited a significant ($p = 0.0118$), poor prognostic role in HCC, whereas a nonsignificant prognostic role was found for other cancers, including breast, cervical, esophagus, and lung cancers (Figure 1C), suggesting a differential prognostic role of *GPR50* in various cancers. Thus, these results indicate that *GPR50* may have an oncogenic role in liver cancer.

We further examined *GPR50* expression in liver cancer using TCGA dataset through the SurvExpress web and confirmed *GPR50* overexpression (Figure 1D). We then examined mutation and copy number alterations (CNAs) in the liver cancer TCGA dataset through the cBioPortal web and found that approximately 3% of the samples showed mutation, amplification, deep deletion, or mRNA upregulation of *GPR50* genes (Figure 1E). Moreover, we checked *GPR50* mRNA and protein expression levels in the normal hepatocyte cell line MIHA and several HCC cell lines, including HepG2, SNU449, and SNU475, via reverse-transcriptase PCR (RT-PCR) and western blot analyses and found that *GPR50* expression was clearly overexpressed in the HCC cell lines (Figure 1F). Overall, these results indicate that *GPR50* expression is dysregulated in various cancers and specifically upregulated in HCC.

GPR50 Knockdown Decreases Cell Proliferation, Migration, Sphere Formation, and Drug Resistance

To examine the role of *GPR50* in HCC, we knocked down *GPR50* using GPR50-targeted short hairpin RNA (shRNA) in two HCC cell lines: HepG2 and SNU475. *GPR50* knockdown was confirmed (approximately 95%) via RT-PCR and western blot analysis (Figure 2A). We found that cell proliferation was decreased upon *GPR50* silencing in both HepG2 and SNU475 cells (Figure 2B). In addition, cell migration, sphere formation, and drug resistance (Figures 2C, 2D, and 2F) were attenuated in GPR50-knockdown HepG2 and SNU475 cells compared with their normal counterparts. We also found that expression of stemness markers, such as *NANOG*, *SOX2*, *OCT4*, and *KLF4*, and drug-resistance markers, such as *P-GP*, *ABCG2*, *ABCC1*, *ALDH1A1*, and *ABCB5*, was decreased upon *GPR50* knockdown (Figures 2E and 2G; Figure S1), suggesting that *GPR50* has oncogenic ability and regulates HCC progression.

GPR50 Is a Novel Regulator of the Notch Signaling Pathway

As *GPR50* knockdown attenuated cancer progression, we attempted to uncover the underlying signaling mechanism. For that, we checked several signaling pathways that potentially contribute to cancer progression. We found that expression of *HES1*, a Notch signaling target gene, was significantly downregulated upon *GPR50* knockdown (Figure 3A). We did not detect any significant differences in the expression of target genes of other signaling pathways, including Wnt, Hedgehog, and Hippo, which are predominantly involved in cancer progression, stemness, and metastasis.[29–33] Expression of other Notch pathway target genes, such as *NOTCH1*, *MAML1*, and *RBPjK*, was also suppressed upon *GPR50* knockdown in HCC cells (Figure 3B). Western blotting confirmed the reduced protein expression levels of Notch intracellular domain (NICD) and HES1 upon *GPR50* knockdown (Figure 3C). Moreover, transcription activity of *HES1* and *HES5* was reduced upon *GPR50* silencing (Figures 3D and 3E).

Next, we induced the Notch signaling pathway in *GPR50*-knockdown cells using NICD to test whether NICD overexpression can relieve *GPR50* suppression effects. FLAG-NICD overexpression in SNU475 cells was confirmed by western blot analysis using a FLAG antibody (Figure 3F). We then analyzed *HES1* and *HES5* promoter activity and found that the shGPR50-induced reduction in *HES1* and *HES5* transcriptional activity was increased upon NICD overexpression (Figures 3G and 3H). Moreover, shGPR50-induced reduction in cell

(LEU; n = 30), liver (HCC; n = 9), lung (LUC; n = 73), lymphoma (LYM; n = 38), melanoma (MEL; n = 12), myeloma (MYE; n = 5), ovarian (OVC; n = 5), pancreatic (PAC; n = 9), prostate (PRC; n = 3), and sarcoma (SAR; n = 17) cancers. (B) Analysis of GEO: GSE1477, GSE7803, GSE20347, GSE45436, and GSE2514 datasets for *GPR50* mRNA expression in BRC (n = 28), CEC (n = 31), ESC (n = 34), HCC (n = 134), and LUC (n = 39) compared with normal breast, cervical, esophageal, liver, and lung tissue. Other GEO datasets for BRC, CEC, ESC, HCC, and LUC cancers were incorporated into Table S1. (C) Kaplan-Meier curves for clinical outcomes of patients with breast (n = 962), cervical (n = 191), esophageal (n = 184), liver (n = 361), and lung (n = 475) cancers, respectively, with high (red) and low (green) expression levels of *GPR50*. (D) *GPR50* mRNA expression in HCC. Boxplot generated by the SurvExpress web shows *GPR50* expression levels and the p value (t test of differences in TCGA RNA sequencing [RNA-seq] dataset). Low-risk (n = 191) and high-risk (n = 190) groups are shown in green and red, respectively. (E) *In silico* examination using cBioPortal reveals that 2.9% of samples had alterations in *GPR50* expression in HCC TCGA PanCan data (n = 348). (F) GPR50 expression was analyzed by RT-PCR and western blotting in the indicated normal hepatic cell line and different HCC cell lines. *GAPDH*/actin were used as a loading control.

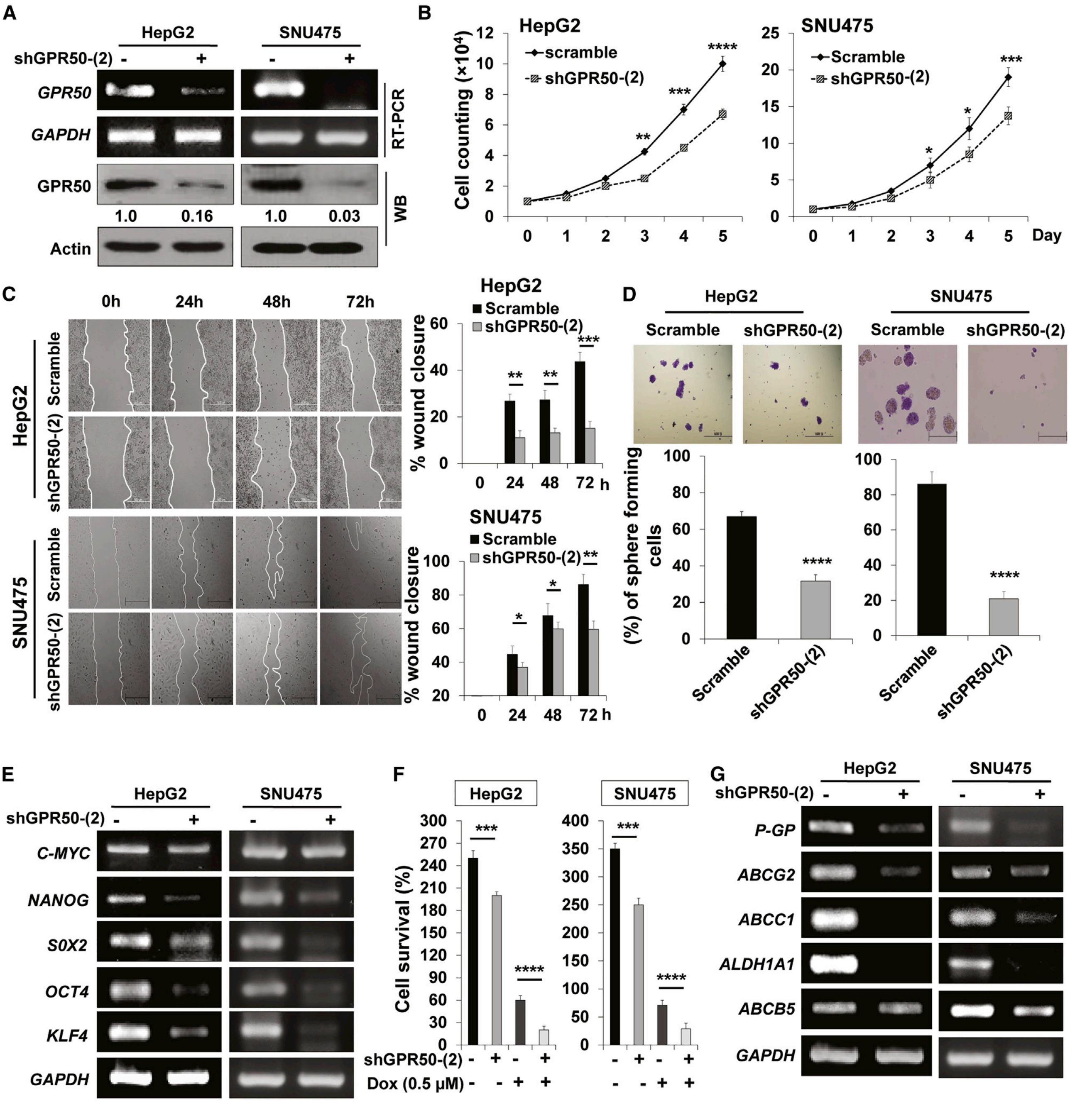

Figure 2. *GPR50* Knockdown Led to Suppressed Cancer Properties in HCC

(A) GPR50 expression in scramble and shGPR50-transduced cells was analyzed by RT-PCR and western blotting in the indicated cancer cell lines. *GAPDH*/actin was used as a loading control. (B) Cell proliferation was analyzed using trypan blue. Cells were counted over 5 days. (C) Wound-healing assay to test migration of the indicated cells. Cell migration was observed at the indicated time points and presented as percentage (%) wound enclosure (right panel). Photos were acquired using inverted light microscopy. (D) Cell-sphere formation assay was performed using noncoated culture dishes. Spheres were counted after 5 days of culture with crystal violet staining and presented as the percent (%) of colonies. Photos were acquired by inverted light microscopy. (E) mRNA expression of stemness markers was analyzed by RT-PCR. *GAPDH* was used as an internal standard. (F) Effect of *GPR50* knockdown on drug resistance was measured by cell counting after 48 h of doxorubicin (DOX) treatment (0.5 μM). (G) mRNA expression levels of drug-resistance marker genes were analyzed by RT-PCR using GPR50-knockdown cells. *$p < 0.05$, **$p < 0.01$, ***$p < 0.001$, ****$p < 0.0001$.

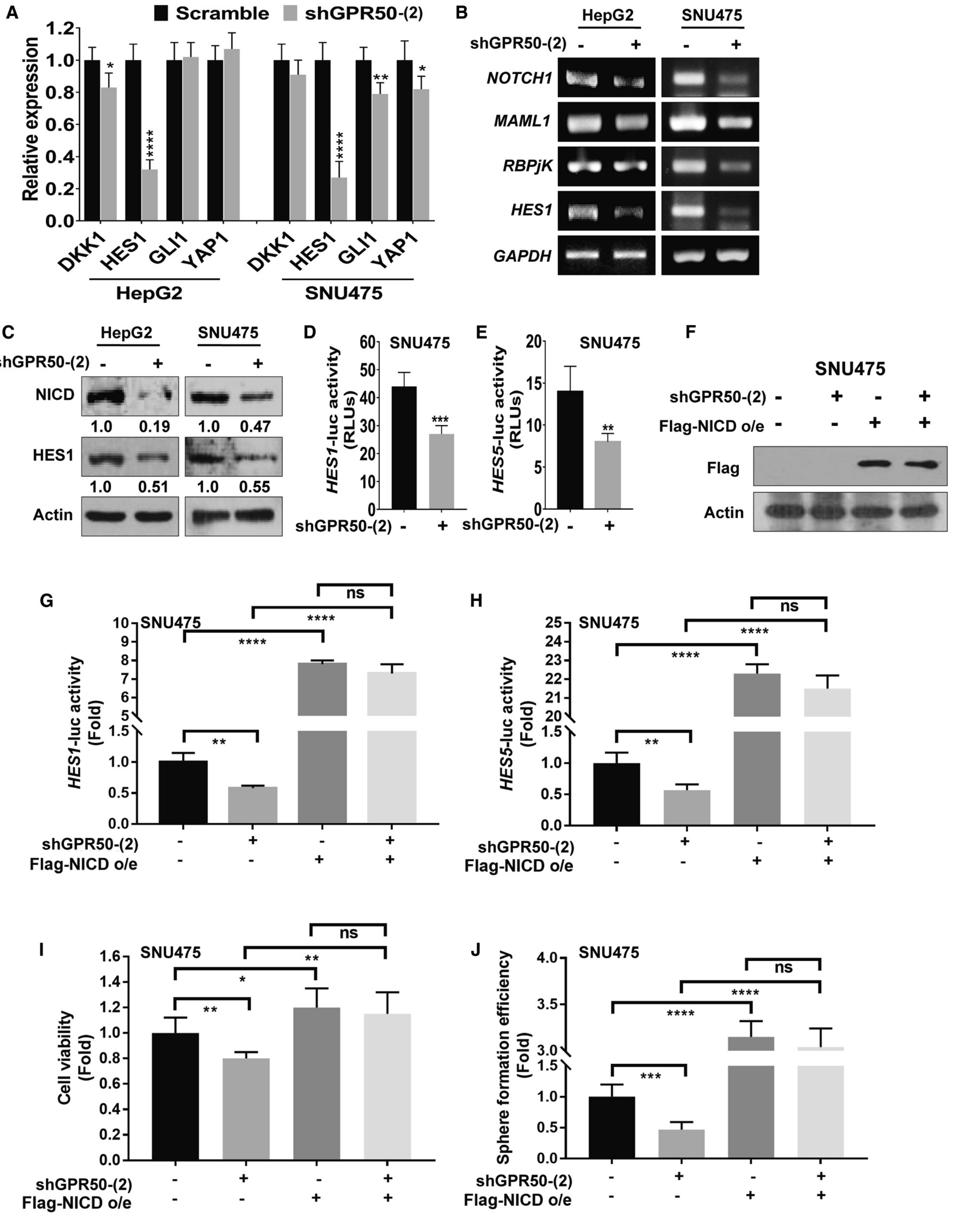

(legend on next page)

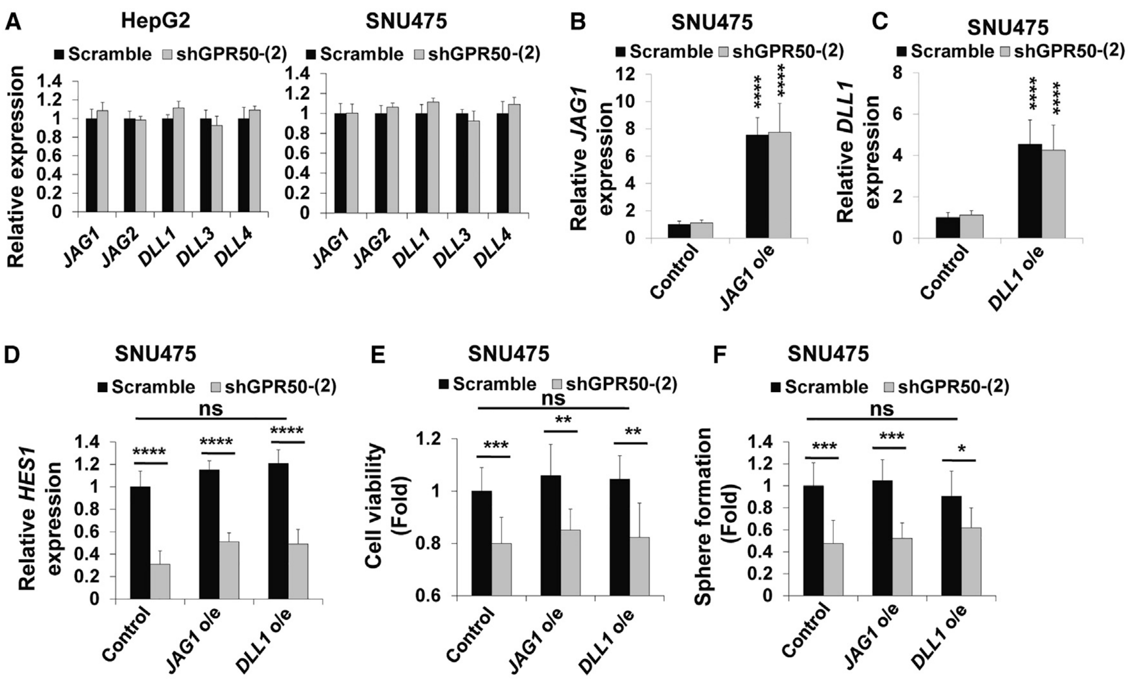

Figure 4. GPR50 Does Not Regulate the Ligand-Dependent Notch Signaling Pathway

(A) mRNA expression levels of the Notch ligands Jagged 1 and 2 (*JAG1* and *JAG2*) and Delta-like 1, 3, and 4 (*DLL1*, *DLL3*, and *DLL4*) were analyzed using real-time RT-PCR and normalized to that of *GAPDH.* (B and C) *JAG1* (B) and *DLL1* (C) overexpression was confirmed by real-time RT-PCR and normalized to that of *GAPDH.* (D–F) *HES1* mRNA expression (D), cell viability (E), and sphere formation (F) were analyzed after overexpression of *JAG1* and *DLL1* in the indicated cells. ns, no significance; *$p < 0.05$, **$p < 0.01$, ***$p < 0.001$, ****$p < 0.0001$.

viability and sphere formation was also reversed upon NICD overexpression (Figures 3I and 3J). These results indicate that attenuation of HCC progression via shGPR50 can be regulated through the Notch signaling pathway.

The *GPR50*-Regulated Notch Signaling Pathway Is Notch Ligand Independent

We further assessed the Notch ligand dependency of the *GPR50*-regulated Notch signaling pathway, because this pathway can be regulated in a ligand-dependent[34–36] or -independent[37] manner. First, we focused on ligand-dependent Notch signaling activation upon *GPR50* knockdown. mRNA expression of the Notch signaling ligands Jagged 1 (*JAG1*) and 2, as well as delta-like ligand 1 (*DLL1*), 3, and 4, was not significantly altered in shGPR50-HepG2 and SNU475 cells compared with their normal counterparts (Figure 4A). To confirm, we overexpressed *JAG1* and *DLL1* in scramble and shGPR50-SNU475 cells, validated by RT-PCR (Figures 4B and 4C), and found that Notch ligand overexpression did not significantly affect the shGPR50-induced reduction in *HES1* mRNA expression, cell viability, and sphere formation (Figures 4D–4F). Taken together, these results indicate that suppression of Notch signaling via *GPR50* knockdown occurs in a ligand-independent manner.

GPR50 Regulates *ADAM17* Transcription through the AKT/SP1 Axis

Next, we investigated the ligand-independent activation of Notch signaling by analyzing mRNA expression levels of ADAM metallopeptidases, including *ADAM9*, *-10*, *-12*, and *-17*, which have been reported to regulate the Notch signaling pathway.[37–41] Among these ADAMs, *ADAM17* mRNA expression was significantly downregulated upon *GPR50* knockdown in both HCC cell lines, HepG2 and SNU475 (Figure 5A). Moreover, we analyzed the correlation between *ADAMs* and *GPR50* mRNA expression in HCC using TCGA database through the cBioPortal web and found that mRNA expression levels of the studied

Figure 3. Silencing of *GPR50* Expression Downregulates Notch Signaling in HCC Cell Lines

(A) mRNA expression levels of different signaling target genes (*DKK1* for Wnt signaling, *HES1* for Notch signaling, *GL1* for Hedgehog signaling, and *YAP1* for Hippo signaling) were analyzed using qRT-PCR and normalized to that of *GAPDH.* (B) mRNA expression levels of *NOTCH1*, *MAML1*, *RBPjK*, and *HES1* were analyzed using RT-PCR; *GAPDH* was used as an internal standard. (C) Protein levels of NICD and HES1 were assessed using western blotting; actin was used as a loading control. (D and E) Notch signaling transcriptional activity was analyzed using *HES1* (D) and *HES5* (E) luciferase assays. (F) Overexpression of FLAG-NICD was confirmed by western blot analysis using FLAG antibody; actin was used as a loading control. (G and H) Fold change of Notch signaling transcriptional activity was analyzed after overexpression of NICD in the indicated cells using *HES1* (G) and *HES5* (H) luciferase assays. (I and J) Cell viability (I) and sphere formation (J) were analyzed after overexpression of NICD in the indicated cells. ns, no significance; *$p < 0.05$, **$p < 0.01$, ***$p < 0.001$, ****$p < 0.0001$.

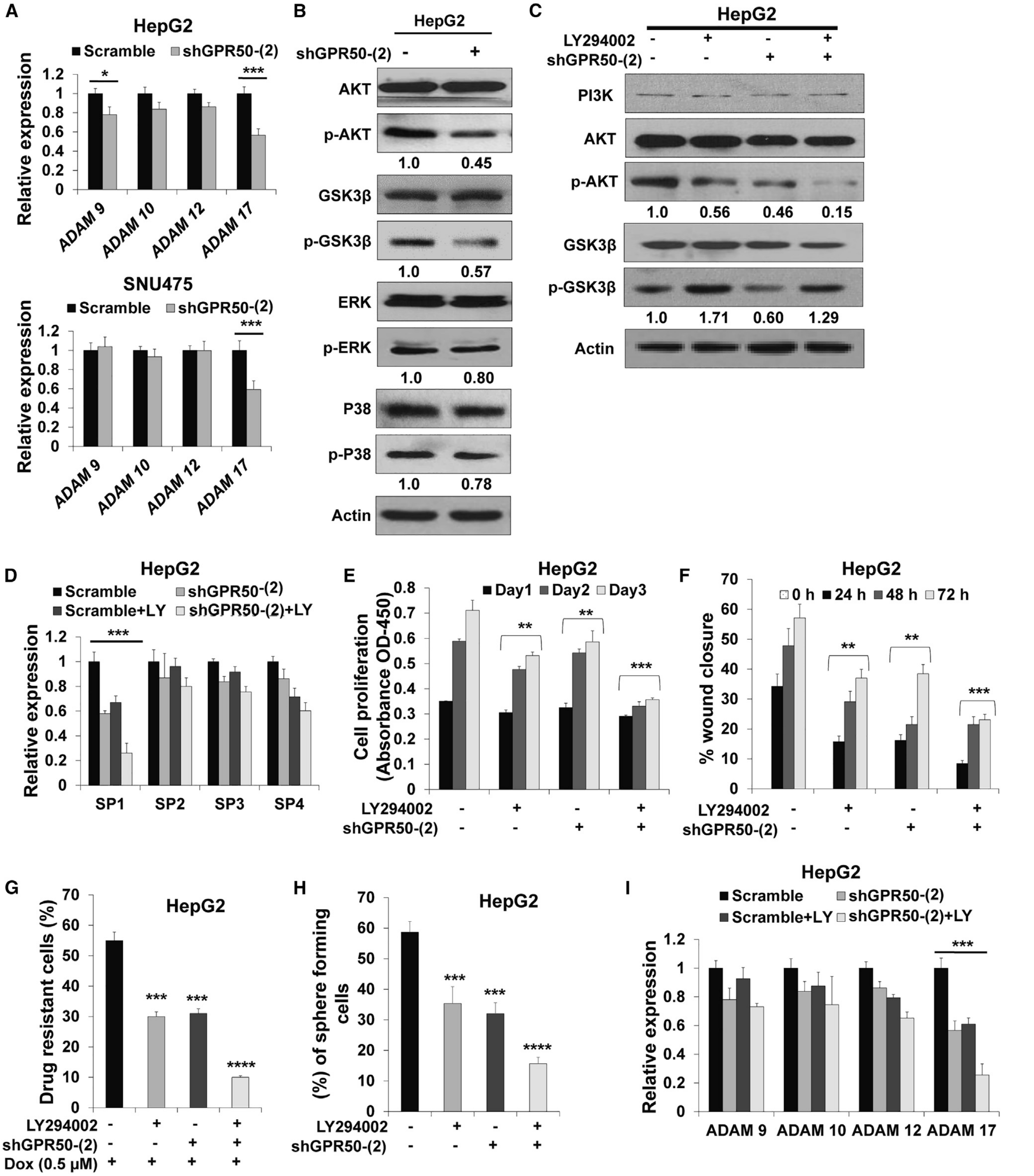

Figure 5. GPR50 Regulates *ADAM17* Transcription via the AKT/SP1 Axis

(A) Expression levels of Notch signaling regulating genes *ADAM9*, *ADAM10*, *ADAM12*, and *ADAM17* were analyzed using qRT-PCR; *GAPDH* was used as an internal standard. (B) Protein expression levels of AKT, phosphorylated (p)-AKT, GSK3β, p-GSK3β, ERK, p-ERK, p38, and p-p38 were analyzed using western blotting; actin was

(legend continued on next page)

ADAMs were positively correlated with *GPR50* expression, with *ADAM17* showing the most significant correlation (Figure S2).

Therefore, we aimed to uncover how GPR50 regulates *ADAM17* transcription by examining whether *GPR50* knockdown affects the AKT, extracellular signal-regulated kinase (ERK)/mitogen-activated protein kinase (MAPK), and p38/MAPK signaling pathways, as previous studies reported that transcription of *ADAM17* can be regulated through these pathways.[42–44] We found that AKT phosphorylation was downregulated in shGPR50-HepG2 cells compared with that in scramble-HepG2 cells, whereas the ERK/MAPK and p38/MAPK signaling pathways were not altered upon *GPR50* knockdown (Figure 5B). Glycogen synthase kinase 3β (GSK3β phosphorylation at Ser9 was also altered upon *GPR50* suppression (Figure 5B). To confirm these findings, we treated *GPR50*-knockdown cells with the AKT inhibitor LY294002 (20 μM).[45] LY294002 treatment drastically reduced AKT phosphorylation in *GPR50*-knockdown cells compared with control cells (Figure 5C). LY294002 treatment also induced GSK3β phosphorylation in sh*GPR50* cells compared with that in control cells (Figure 5C), which may subsequently regulate other signaling pathways, including GSK3β/mammalian target of rapamycin (mTOR) or Wnt/β-catenin, which are yet to be investigated.

As the *ADAM17* promoter region contains a high guanine-cytosine (GC)-rich sequence that was depicted to bind various transcription factors, including SP1,[46,47] we tested whether *GPR50* silencing regulates *SP1* expression. We examined the mRNA expression levels of all SP family genes (*SP1*, *-2*, *-3*, and *-4*) and found that *SP1* expression was significantly downregulated upon *GPR50* suppression (Figure 5D). Moreover, *SP1* expression was significantly attenuated upon LY294002 treatment in *GPR50*-knockdown cells compared with that in control cells (Figure 5D). Cell proliferation, wound healing/migration, drug resistance, and sphere formation (Figures 5E–5H) were also widely attenuated upon LY294002 treatment. We also analyzed ADAM expression and found that *ADAM17* was significantly downregulated upon LY294002 treatment in *GPR50*-silenced cells compared with that in control cells (Figure 5I). Thus, these results support the notion that *GPR50* knockdown mediates *ADAM17* downregulation through the AKT/SP1 axis.

GPR50 Directly Interacts with ADAM17 and Regulates ADAM17 Activity

As *GPR50* knockdown suppressed *ADAM17* transcription, we analyzed ADAM17 protein levels upon *GPR50* silencing and found that ADAM17 protein levels were attenuated in sh*GPR50* cells (Figure 6A). ADAM17 is a proteolytic enzyme that can cleave Notch receptors and subsequently activate the Notch signaling pathway.[48] Thus, we examined ADAM17 activity in *GPR50*-knockdown cells and found reduced ADAM17 activity (Figure 6B). We theorized that GPR50 and ADAM17 can directly interact with each other (as both are cell membrane proteins), which was confirmed via coimmunoprecipitation (coIP; Figure 6C), indicating that ligand-independent Notch signaling activation is mediated through the GPR50-ADAM17 interaction.

Overexpression of *ADAM17* Relieves *GPR50*-Knockdown Effects

We next overexpressed ADAM17 in *GPR50*-knockdown SNU475 cells to test whether *ADAM17* overexpression can relieve the effects of shGPR50. Overexpression of FLAG-*ADAM17* was confirmed by western blot analysis using a FLAG antibody (Figure 6D). Afterward, Notch signaling activation upon *ADAM17* overexpression in *GPR50*-knockdown cells was analyzed by examining *HES1* and *HES5* transcription activity via luciferase assays (Figures 6E and 6F). shGPR50-induced suppression of *HES1* and *HES5* transcription activity was reversed upon *ADAM17* overexpression (Figures 6E and 6F). Moreover, treatment with marimastat (4 μM),[49] an ADAM17 inhibitor, suppressed the effects of *ADAM17* overexpression specially in shGPR50-SNU475 cells (Figures 6E and 6F). Similarly, shGPR50-induced reduction in ADAM17 activity was relieved upon *ADAM17* overexpression in SNU475 cells; conversely, marimastat (4 μM) treatment of *ADAM17*-overexpressed SNU475 cells reduced ADAM17 activity, which was marked in shGPR50-SNU475 cells (Figure 6G). Moreover, the shGPR50-induced reduction in cell viability (Figure 6H) and sphere formation (Figure 6I) was rescued upon *ADAM17* overexpression in SNU475 cells, whereas marimastat alone or marimastat + shGPR50 suppressed the effects of *ADAM17* overexpression in SNU475 cells. These results indicate that the shGPR50-induced suppression of HCC progression is mediated through ADAM17-dependent Notch signaling suppression.

Overexpression of *GPR50* Induces Cancer Progression through ADAM17-Dependent Notch Signaling Pathway Activation

Finally, we assessed the role of the ADAM17-activated Notch signaling pathway during *GPR50* overexpression in HCC. We first confirmed *GPR50* overexpression in the MIHA and HepG2 cell lines using RT-PCR and western blot analysis (Figure 7A) and then analyzed the effect of *GPR50* overexpression in MIHA and HepG2

used as a loading control. (C) Scramble and shGPR50-HepG2 cells were treated with/without LY294002 (20 μM), and then protein levels of PI3K, AKT, p-AKT, GSK3β, and p-GSK3β were assessed by western blotting; actin was used as a loading control. (D) Scramble and shGPR50-HepG2 cells were treated with LY294002 (20 μM), and then mRNA expression levels of *SP1*, *SP2*, *SP3*, and *SP4* were analyzed using qRT-PCR and normalized to that of *GAPDH*. (E and F) Scramble and shGPR50-HepG2 cells were treated with LY294002 (20 μM), and then cell viability (E) and wound healing (F) were analyzed at the indicated time points. (G) Scramble and shGPR50-HepG2 cells were treated with LY294002 (20 μM) in the presence of DOX (0.5 μM), and then drug resistance was measured by cell counting after 48 h. (H) Scramble and shGPR50-HepG2 cells were treated with LY294002 (20 μM), and then a sphere-formation assay was performed using the noncoated culture dishes. Spheres were counted after 5 days of culture using crystal violet staining and represented as the percent (%) of colonies. (I) Scramble and shGPR50-HepG2 cells were treated with LY294002 (20 μM) and then mRNA expression levels of *ADAM9*, *ADAM10*, *ADAM12*, and *ADAM17* were analyzed using qRT-PCR and normalized to that of *GAPDH*. *$p < 0.05$, **$p < 0.01$, ***$p < 0.001$, ****$p < 0.0001$.

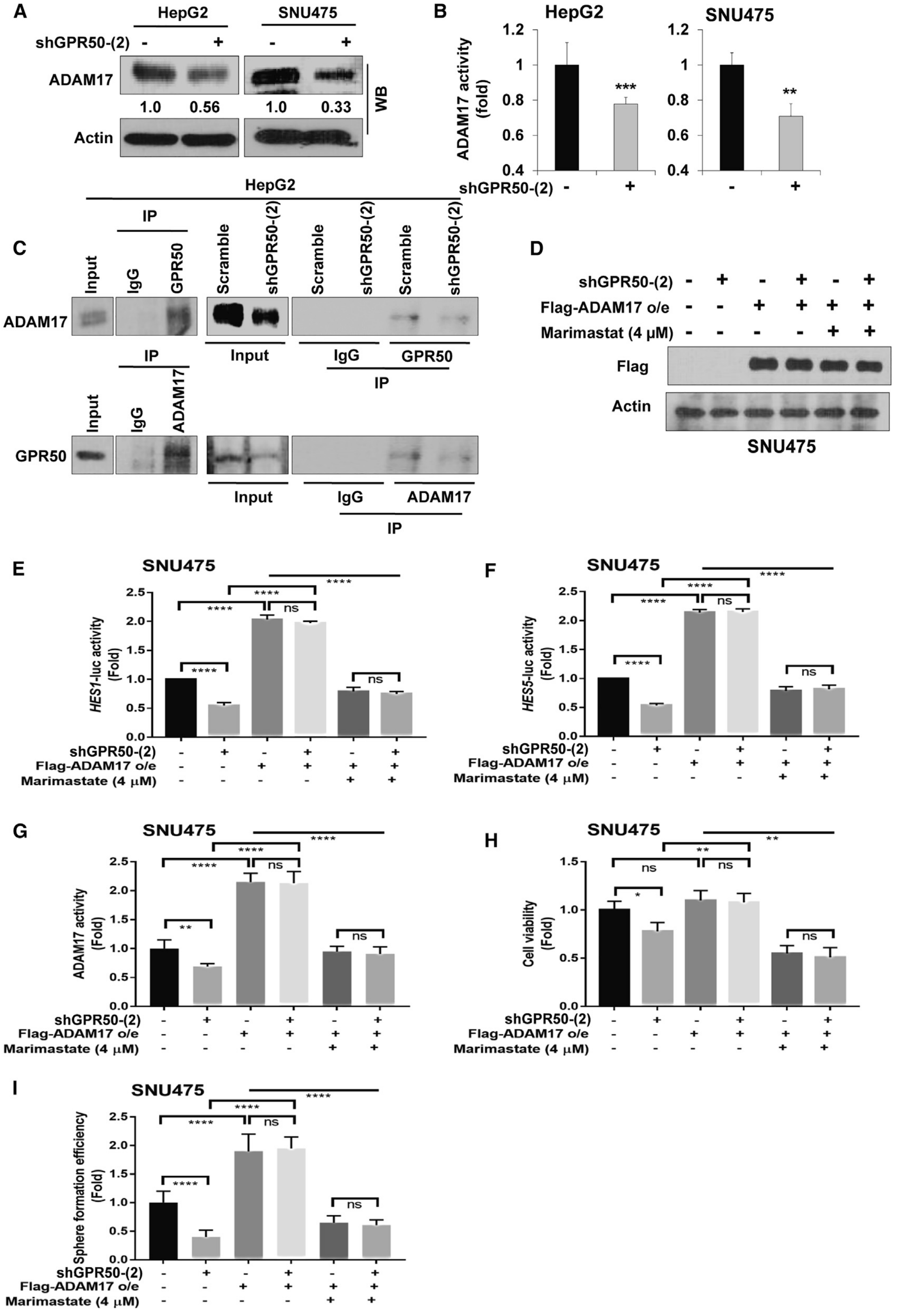

(legend on next page)

cells. Cell viability was found significantly increased in both MIHA and HepG2 cells upon *GPR50* overexpression (Figure 7B); similarly, colony-formation capability was also significantly induced (Figure 7C). Next, we evaluated whether *GPR50* overexpression upregulates ADAM17 and the expression of Notch signaling-responsive proteins, such as NICD and HES1. Expectedly, ADAM17, NICD, and HES1 protein levels were upregulated upon *GPR50* overexpression in MIHA and HepG2 cells (Figure 7D). Subsequently, transcriptional activity of the Notch target genes *HES1* and *HES5* was significantly enhanced upon *GPR50* overexpression in both MIHA and HepG2 cells (Figures 7E and 7F). Moreover, ADAM17 activity upon *GPR50* overexpression in HepG2 cells was significantly augmented compared with that of control HepG2 cells (Figure 7G).

To confirm the GPR50-induced regulation of the Notch signaling pathway through ADAM17, we treated GPR50-overexpressed HepG2 cells with marimastat (4 μM) and/or the Notch signaling (γ-secretase) inhibitor DAPT (20 μM).[50] GPR50 protein expression was not significantly altered upon marimastat and/or DAPT treatment in *GPR50*-overexpressed HepG2 cells, whereas the *GPR50* overexpression-induced upregulation of ADAM17, NICD, and HES1 was dramatically relieved upon marimastat and/or DAPT treatment (Figure 7H). Moreover, the *GPR50* overexpression-induced ADAM17 activity was significantly attenuated upon marimastat treatment (Figure 7I), whereas enhanced *HES1* and *HES5* transcriptional activity was significantly relieved upon treatment with marimastat and/or DAPT in HepG2 cells (Figures 7J and 7K). These results strongly suggest that GPR50 regulates HCC progression through the ADAM17-mediated Notch signaling pathway.

DISCUSSION

An orphan GPCR (GPR50) was shown to regulate bipolar-affective disorder, lipid metabolism, thermogenesis, adipogenesis, and neuronal development,[21–25] although it has been claimed that GPR50 has high sequence similarity with melatonin receptors (MT1 and MT2), but melatonin does not bind to GPR50.[19,20] GPR50 was also reported to interact directly with various proteins, such as TIP60, NOGO-A, MT1, and MT2, via heterodimerization through the large carboxyl-terminal tail (C-tail) of GPR50.[24,51,52] Moreover, previous studies displayed that GPR50 can directly interact with transforming growth factor-β1 (TGF-β1) and constitutively activate the canonical SMAD2/3 signaling pathway, which contributes to regulation of breast cancer progression, indicating that GPR50 can act as a tumor suppressor in breast cancer.[27,28] In addition, our previous study was given a clue that several GPCRs, including GPR50, are involved in the reprogramming of somatic cells to cancer stem cells and in the maintenance of stemness function.[26] However, little is known regarding the biological significance of GPR50 in liver cancer.

Here, we investigated the expression patterns of GPR50 in various cancer cells and found dysregulated GRP50 expression. Moreover, GPR50 expression was lower in normal hepatocytes than in HCC cells. We also found that *GPR50* knockdown attenuates cell proliferation, sphere formation, migration, and drug resistance of HCC cells, whereas overexpression of *GPR50* upregulates cancer properties in hepatocytes (see Figures 2 and 7), strongly supporting an oncogenic function for GPR50 in HCCs. We further investigated the underlying molecular mechanisms of GPR50-mediated HCC regulation and found that GPR50 regulates HCC cell proliferation, migration, sphere formation, and drug resistance via the Notch signaling pathway. We identified, for the first time, a novel GPR50-mediated Notch signaling activation pathway that is activated in a ligand-independent manner.

Notch signaling is involved in most multicellular processes in cancer cells, including cell fate, proliferation, metastasis, invasion, and stemness.[53–57] Recent studies have depicted that the Notch signaling pathway can play either pro-oncogenic or tumor-suppressive roles in cancer cells.[58–60] Notch signaling is normally triggered by a Notch-activating ligand that is subsequently processed through two proteolytic cleavage events via ADAM17 and the γ-secretase complex.[48] This cleavage can then result in release of the NICD, which is imported to the nucleus and binds to cotranscription factors, including MAML1, RBP-J, p300, and CSL, to trigger expression of Notch signaling target genes.[53,54] Notably, the Notch signaling pathway is significantly associated with liver cirrhosis and HCC.[61] Although various members of the Notch signaling pathway may act as inhibitors or enhancers of HCC, the Notch signaling pathway generally plays a carcinogenic role in HCC, and activation of Notch signaling has been associated with a more malignant phenotype.[62] Therefore, identification of reliable Notch pathway regulatory mechanisms is critical for the application of Notch-based HCC therapeutic strategies, as regulation of the Notch pathway can potentially suppress HCC progression and aggressiveness.

Figure 6. GPR50 Directly Interacts with ADAM17 and Activates the Ligand-Independent Notch Signaling Pathway via ADAM17 in HCC

(A) Protein expression levels of ADAM17 were analyzed using western blotting; actin was used as a loading control. (B) Fold change in ADAM17 activity was analyzed using the ADAM17 ELISA assay. (C) Lysates from the indicated cells were used for immunoprecipitation using Protein A/G Sepharose, as well as antibodies specific for GPR50, ADAM17, and normal IgG. The immunoprecipitates were analyzed by western blotting with the indicated antibodies. For inputs, lysates were analyzed by western blotting with the indicated antibodies. (D) Overexpression of FLAG-*ADAM17* was confirmed by western blot analysis using FLAG antibody; actin was used as a loading control. (E and F) Fold change in Notch signaling transcriptional activity was analyzed using *HES1* (E) and *HES5* (F) luciferase assays after overexpression of *ADAM17* and treatment with/without marimastat (4 μM). (G) Fold change in ADAM17 enzymatic activity was analyzed using the ADAM17 ELISA assay after overexpression of ADAM17 and treatment with/without marimastat (4 μM). (H) Cell viability was analyzed using the EZ-cytox 4-[3-(4iodophenyl)-2-(4- nitrophenyl)-2H-5-tetrazolio]-1,3-benzene disulfonate (WST-1) assay after overexpression of ADAM17 and treatment with/without marimastat (4 μM) after 24 h. (I) Sphere formation assay was performed using the noncoated culture dishes after overexpression of ADAM17 and treatment with/without marimastat (4 μM). Spheres were counted after 5 days of culture using crystal violet staining and presented as fold change in colonies. ns, no significance; **p < 0.01, ****p < 0.0001.

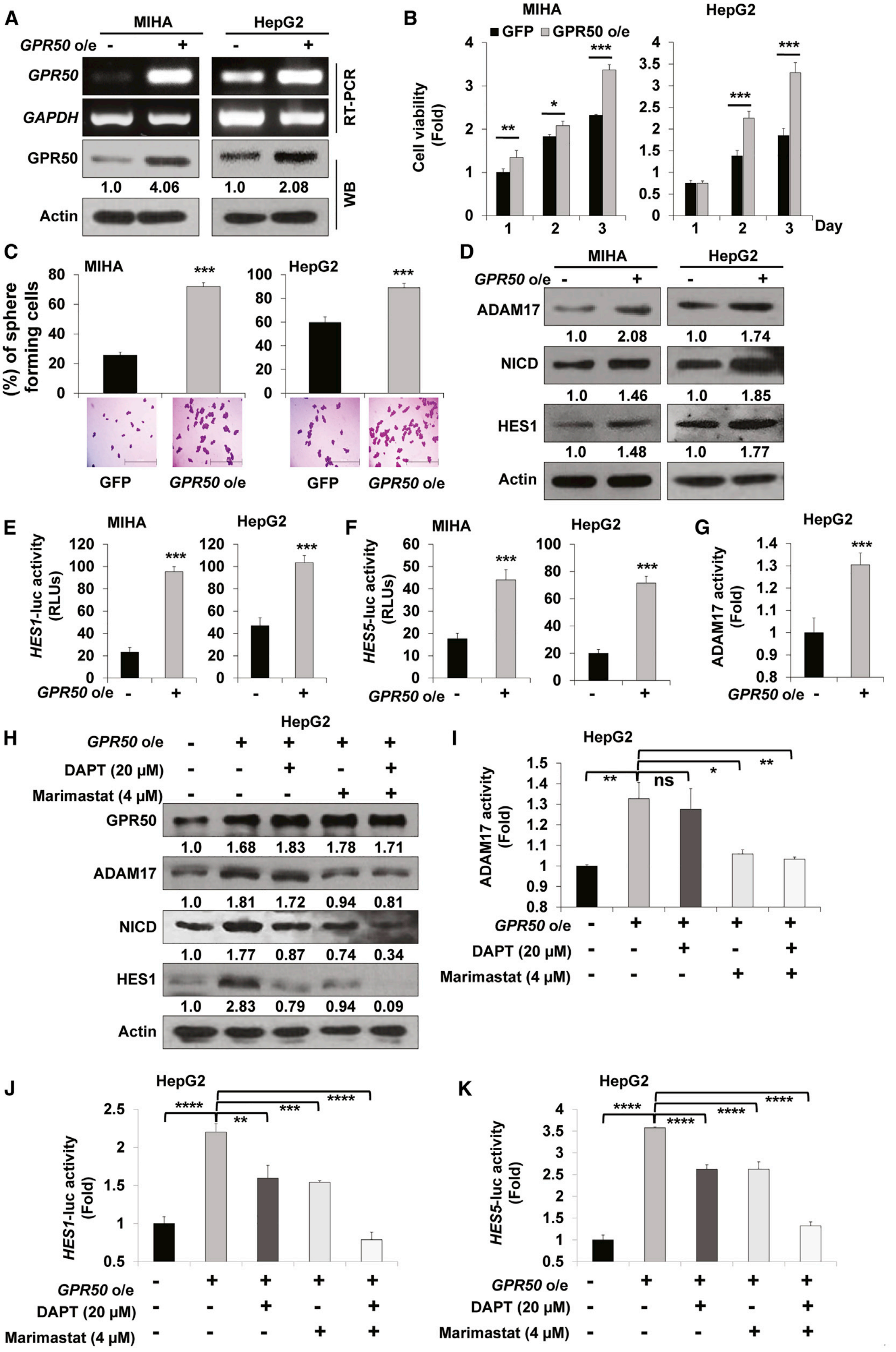

(legend on next page)

We further found that the GPR50-regulated Notch signaling pathway is mediated by ADAM17, independent from the Notch ligands JAG and DLL. ADAM, a single-pass transmembrane protein, is involved in multiple cellular functions, such as migration, proteolysis of extracellular matrix (ECM) components, and shedding of membrane proteins (e.g., cytokines and growth factors); fertilization; development; inflammation; asthma; and neurodegenerative diseases, such as Alzheimer's disease.[63–65] Several ADAM family proteins have been identified and are characterized by their domain organization, including a pro-domain; a metalloprotease, disintegrin, cysteine-rich, epidermal growth factor (EGF)-like domain; and transmembrane domains, and a C-terminal cytoplasmic tail. Several reports have demonstrated that ADAMs play an important role in HCC pathogenesis.[66] ADAM17, also known as TACE (tumor necrosis factor α [TNF-α]-converting-enzyme), has been reported to be a Notch receptor molecular scissor, which leads to tumorigenesis and tumor progression.[67,68] Moreover, ADAM17 activates the Notch signaling pathway in a ligand-independent manner[37] and is reported to regulate cell proliferation, angiogenesis, invasion, and apoptosis of cancer cells by regulating Notch signaling.[38,39,69] However, ligand-independent Notch signaling activation via ADAM17 is not fully elucidated.

Consistent with our results, a number of studies depicted that ligand-independent Notch activation is mediated by ADAM17.[37,70] Another study demonstrated Notch signaling activation without cell-cell contact in the presence of soluble JAG1.[34] Moreover, a study demonstrated that several GPCRs, including orphan GPCRs, resulted in ADAM17 activation and subsequently induced TGF-α shedding in HEK293 cells.[71] Furthermore, GPCRs, including GPR50, were reported to interact with other receptors from the same family or different receptor and transporter proteins through heterodimerization, eventually forming molecular complexes.[24,52,72] Similarly, our findings demonstrated that GPR50 can form a molecular complex with ADAM17 through direct interaction in HCC (see Figure 6C), which subsequently activates Notch signaling via ADAM17 activity, strongly supporting a novel signaling pathway between GPR50 and the ligand-independent, ADAM17-mediated Notch signaling.

We further demonstrated that GPR50 can regulate ADAM17 transcription and translation through the AKT/SP1 signaling axis, which is in agreement with previous studies that ADAM17 can be transcriptionally regulated via the AKT, ERK/MAPK, and p38/MAPK signaling pathways.[42–44] Moreover, the ADAM17 promoter has a high GC-rich sequence where a number of transcription factors, including SP1, can bind.[46,47] SP1 has been shown to have a potential role in cancer by regulating the transcription of several genes that have high GC-rich sequences in their DNA-binding promoter regions.[73–76] SP1 is regulated by its upstream effectors, including the phosphatidylinositol 3-kinase (PI3K)/AKT, ERK, and p38/MAPK signaling pathways.[77–80] Moreover, a study demonstrated that GPCRs can regulate SP1 via their downstream proteins and effectors (i.e., β-arrestin 1), which represses leukemic cell senescence.[81] Therefore, by taking into consideration these previous studies and our findings, there is ample evidence supporting GPR50-mediated ADAM17 transcription and translation via the AKT/SP1 axis.

In conclusion, we demonstrated how the orphan receptor GPR50 regulates the ligand-independent activation of Notch signaling through GPR50-mediated modulation of ADAM17 activity in HCC (Figure 8). A signaling cascade initiating from GPR50 was uncovered, wherein GPR50 was also found to regulate ADAM17 transcription and translation via the AKT/SP1 axis in HCC (Figure 8). Thus, our findings revealed the molecular basis underlying the GPR50 and ADAM17 complex-mediated, ligand-independent modulation of the Notch signaling pathway, which can be exploited in Notch-based HCC therapeutic strategies.

MATERIALS AND METHODS

Bioinformatic Analysis

The expected expression levels of the *GPR50* gene in various cancers were retrieved from the Oncomine database (https://www.oncomine.org/resource/login.html).[82,83] Fold changes in mRNA expression in cancer tissues compared with their normal counterparts were acquired using a default threshold. Gene expression was also analyzed using GEO, a web database that gathers submitted high-throughput gene-expression data of chips, microarray, or next-genome sequencing (https://www.ncbi.nlm.nih.gov/geo/).[84] Microarray datasets with accession numbers GEO: GSE1477, GSE7803, GSE20347, GSE45436, and GSE2514 containing gene-expression information of clinical human BRC, CEC, ESC, HCC, and LUC patients, respectively, were used in this study. The other GEO datasets are listed in Table S1. The raw data were retrieved and replotted using GraphPad Prism 7 software (GraphPad Software, La Jolla, CA, USA).

With the use of a web-based tool for survival analysis, SurvExpress (http://bioinformatica.mty.itesm.mx:8080/Biomatec/SurvivaX.jsp),[85]

Figure 7. Overexpression of *GPR50* Augments Notch Signaling via the Ligand-Independent ADAM17 Axis in HCC Cells

(A) *GPR50* overexpression was confirmed by RT-PCR and western blot analysis and normalized to that of *GAPDH* or actin. (B) Cell viability was analyzed over 3 days after overexpression of *GPR50* using the EZ-cytox WST-1 assay. (C) Cell-sphere formation assay was performed using the noncoated culture dishes after *GPR50* overexpression. Spheres were counted after 5 days of culture using crystal violet staining and presented as the percent (%) of colonies. Photos were acquired by inverted light microscopy. (D) Protein expression levels of ADAM17, NICD, and HES1 were analyzed by western blot analysis after *GPR50* overexpression. (E and F) Notch signaling transcriptional activity was analyzed after *GPR50* overexpression using the *HES1* (E) and *HES5* (F) luciferase assays. (G) Fold change in ADAM17 enzymatic activity was analyzed using the ADAM17 ELISA assay after *GPR50* overexpression. (H) Protein expression levels of GPR50, ADAM17, NICD, and HES1 were analyzed by western blot analysis after *GPR50* overexpression and treatment with/without DAPT (Notch signaling inhibitor) and/or marimastat (ADAM17 inhibitor). (I) Fold change in ADAM17 activity was analyzed using the ADAM17 ELISA assay after *GPR50* overexpression and treatment with/without DAPT and/or marimastat. (J and K) Notch signaling transcriptional activity was analyzed after *GPR50* overexpression and treatment with/without DAPT and/or marimastat using the *HES1* (J) and *HES5* (K) luciferase assay. ns, no significance; *p < 0.05, **p < 0.01, ***p < 0.001, ****p < 0.0001.

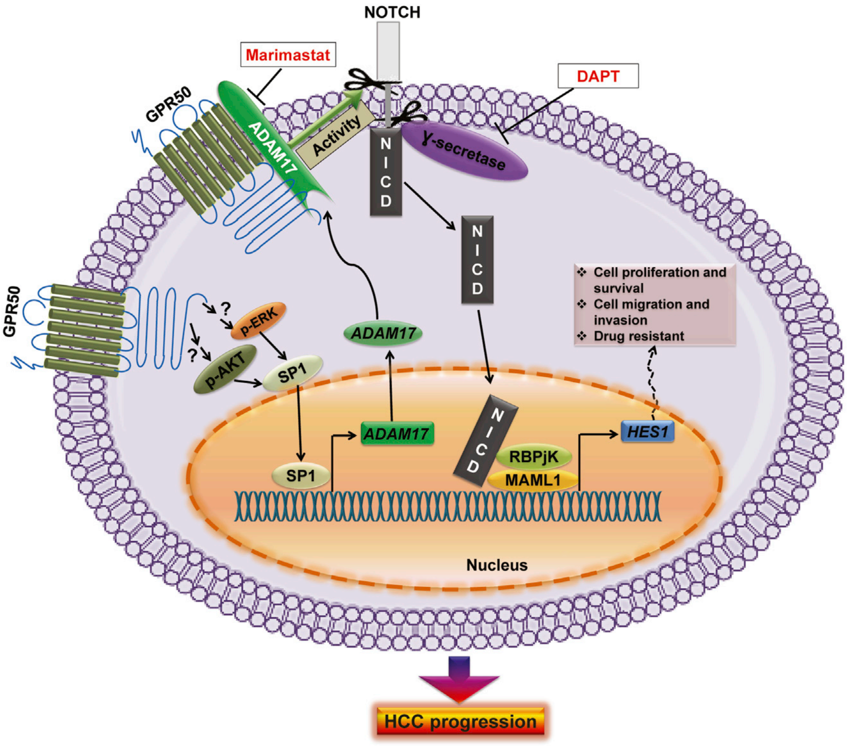

Figure 8. Schematic Diagram Illustrating GPR50 Function in the Notch Signaling Pathway in HCC

GPR50 regulates *ADAM17* transcription via the AKT/SP1 axis and directly interacts with ADAM17 to induce ADAM17 enzymatic activity, which subsequently activates ligand-independent Notch signaling, mediating HCC progression.

we conducted an integrative analysis of *GPR50* mRNA expression levels and clinical outcomes. This database contains more than 39,000 samples and 225 datasets covering tumors from more than 26 different tissues. With the use of this platform, survival plots were generated for GPR50 in specific cancer types using TCGA data. We further performed an integrative analysis of GPR50 and clinical characteristics using cBioPortal, an open-access resource found at http://www.cbioportal.org/,[86,87] which currently provides access to data from more than 48,668 tumor samples and 172 cancer studies in TCGA pipeline. The query interface, combined with customized data storage, enabled us to explore genetic alterations interactively across samples curated from national and international cancer studies for specific genes. The primary search parameters included alterations (amplification, deep deletion, and missense mutations), CNAs, gene-gene correlation from Genomic Identification of Significant Targets in Cancer (GISTIC), and RNA-seq data using the default settings.

Cell Culture

The human HCC cell lines (HepG2, SNU449, and SNU475; American Type Culture Collection [ATCC], Manassas, VA, USA) and the normal hepatocyte cell line MIHA (ATCC) were cultured in DMEM (for MIHA and HepG2 cell lines; Sigma-Aldrich, St. Louis, MO, USA) or RPMI 1640 (for SNU449 and SNU475 cell lines; Sigma-Aldrich), supplemented with 10% heat-inactivated fetal bovine serum (FBS; GE Healthcare HyClone, Pittsburgh, PA, USA), 100 U/mL penicillin (GE Healthcare HyClone), and 100 mg/mL streptomycin (GE Healthcare HyClone). Cells were cultured at 37°C in a humidified atmosphere of 5% CO_2. All cell lines were tested for possible mycoplasma contamination using the *BioMycoX* Mycoplasma PCR Detection Kit (CS-D-25; Cellsafe, Suwon, Republic of Korea) and were authenticated using short tandem repeat (STR) profiling.

GPR50 Knockdown Using shRNA Constructs

Sense and antisense oligonucleotides were synthesized for control (scramble) or *GPR50* knockdown (shGPR50-1 and -2); the sequences are listed in Table S2. The oligonucleotides were then annealed and cloned into a pGreenPuro lentiviral vector (System Biosciences, Mountain View, CA, USA) containing *Bam*HI and *Eco*RI restriction enzyme sites, according to the manufacturer's instructions. Briefly, two oligonucleotides were annealed at 95°C for 2 min in a heat block with annealing buffer, and then the samples were left to cool down to room temperature. The annealed, double-stranded DNA was then ligated into a lentiviral vector using the T4 DNA ligase enzyme (Promega, Madison, WI, USA). Sequences of the newly constructed plasmids were confirmed by sequencing analysis.

Overexpression of *GPR50*, *JAG1*, *DLL1*, *NICD*, and *ADAM17*

For overexpression of *GPR50*, the pGEM-T Easy vector (Promega) was used to clone the complete GPR50 coding sequence using the primers listed in Table S2. Afterward, the complete coding sequence (without the termination codon) was further subcloned into the pCDH-EF1-MCS-T2A-copGFP lentiviral vector (System Biosciences) using *Xba*I and *Eco*RI restriction enzymes and the primers listed in Table S2.

For overexpression of *JAG1*, *DLL1*, *NICD*, and *ADAM17*, cells were incubated overnight to a cell density of 2×10^5 cells per well in a 24-well culture plate. The cells were then transfected with the expression vectors for hemagglutinin (HA)-*JAG1*, GFP-*DLL1*, FLAG-*NICD* (kind gifts from Professor Hee-Sae Park, Chonnam National University, Republic of Korea), and FLAG-*ADAM17* (plasmid number 31713; Addgene, Watertown, MA, USA)[88] using the HyliMax transfection reagent in a 1:3 ratio (Dojindo, Kumamoto, Japan), according to manufacturer's instructions. After 48 h of transfection, the transfected cells were ready for use in further experiments.

Lentivirus Production and Transduction

To generate the lentivirus, the Rev response element (RRE)/REV lentivirus expressing system[89] was used. Briefly, 60%–70% confluent

HEK293T cells were cultured in 100 mm dishes on the day of transfection using the calcium phosphate transfection method. The medium was replaced with fresh medium and plasmids (RRE, REV, and target), after which, the calcium phosphate mixture was added dropwise into the dishes. After 12–16 h, the medium containing the plasmids was removed, and the cells were washed once with PBS. Then, an equal amount of medium was added. After 48 h, the cell supernatant (virus soup) was collected and filtered through a 0.45-μm pore capsule and used for infection, as previously described.[90] Virus titers were also quantified as previously described.[91] For virus infection, we used ~8.0 × 10^8 IU/mL viral particles for stable knockdown in the indicated cells. All experiments were started 72 h postinfection.[92]

RNA Extraction and Quantitative RT-PCR (qRT-PCR)

Total RNA was extracted using an Easy-Blue RNA Extraction Kit (iNtRON Biotechnology, Seongnam, Republic of Korea), and the purified total RNA (2 μg) was reverse transcribed into cDNA using a cDNA synthesis kit (Promega), according to the manufacturer's instructions. For PCR analysis, 1 μL of synthesized cDNA, specific forward and reverse primers, and r-Taq Plus Master Mix (Elpis Biotech, Daejeon, Republic of Korea) were mixed and analyzed by thermocycler PCR, after which, the PCR products were analyzed by agarose gel electrophoresis. DNA was stained via ethidium bromide (EtBr), observed under UV light, and imaged. The images were then analyzed in Photoshop CS6 (Adobe, Mountain View, CA, USA), and the relative expression fold changes were measured using ImageJ; the housekeeping gene glyceraldehyde 3-phosphate dehydrogenase (*GAPDH*) was used as a loading control, as described previously.[90] qRT-PCR was performed using a thermal cycler (PTC-200) with a Chromo4 optical detector (MJ Research; Bio-Rad Laboratories, Hercules, CA, USA) using Fast 2X SYBR Green Master Mix (Applied Biosystems, Foster City, CA, USA); *GAPDH* was used as an internal control, as described previously.[93] The primers used are listed in Table S3.

Western Blotting

Total cell lysates were extracted from the indicated cells using lysis buffer. The cell lysates were then incubated at 4°C for 15 min, vortexed every 2–3 min, and centrifuged at 16,600 × *g* for 15 min. Afterward, concentrations of the extracted proteins were measured using a Bradford assay kit (Bio-Rad Laboratories). Protein samples (40–60 μg) were then loaded onto either 10% or 12% SDS-PAGE gels and electrophoresed, after which, the proteins were transferred to nitrocellulose blotting membranes. The membranes were blocked with 5% skim milk for 1 h and subsequently incubated with primary antibodies against GPR50 (#14032S, 1:1,000; Cell Signaling Technology, Danvers, MA, USA), NANOG (SC-293121, 1:200; Santa Cruz Biotechnology, Dallas, TX, USA), OCT4 (SC-9081, 1:200; Santa Cruz Biotechnology), ABCG2 (SC-377176, 1:1,000; Santa Cruz Biotechnology), phospho-p38 (#9211S, 1:1,000; Cell Signaling Technology), NICD (ab83232, 1:1,000; Abcam, Cambridge, UK), FLAG (F3165, 1:2,000; Sigma-Aldrich), PI3K (SC1637, 1:1,000; Santa Cruz Biotechnology), AKT (SC-1619, 1:1,000; Santa Cruz Biotechnology), phospho-AKT (SC-16646-R, 1:1,000; Santa Cruz Biotechnology), ERK (SC-153, 1:1,000; Santa Cruz Biotechnology), phospho-ERK (SC-7383, 1:1,000; Santa Cruz Biotechnology), p38 (SC-7149, 1:1,000; Santa Cruz Biotechnology), GSK3β (SC-9166, 1:1,000; Santa Cruz Biotechnology), phospho-GSK3β (SC-11757, 1:1,000; Santa Cruz Biotechnology), HES1 (SC-13844, 1:500; Santa Cruz Biotechnology), actin (SC-1616, 1:1,000; Santa Cruz Biotechnology), and α-tubulin (SC-32293, 1:1,000; Santa Cruz Biotechnology) at 4°C overnight. The membranes were then washed three times at 10-min intervals with Tris-buffered saline and Tween 20 (TBS-T; 1,000:1) buffer and incubated with secondary antibodies, including anti-mouse (SC-2005), -goat (SC-2020), or -rabbit (SC-2004) immunoglobulins (Igs) tagged with horseradish peroxidase (HRP) at room temperature for 1 h. Next, after washing with TBS-T buffer for 30 min, the immunoreactive proteins were visualized using an enhanced chemiluminescence (ECL) detection kit (Amersham Bioscience, Piscataway, NJ, USA), as previously described.[90,93–95]

Cell Proliferation and Viability Assays

For cell proliferation analysis, control (scramble-transduced), GPR50-knockdown, or GPR50-overexpressing cells (2 × 10^4 cells/well) were seeded onto 12-well plates. Cells were counted, starting from 24 h up until day 5 using a trypan blue kit. For cell viability assays, cells were seeded onto 96-well plates, and at the indicated time points, EZ-cytox WST-1 reagent (DoGen, Seoul, Republic of Korea) was added at a ratio of 1:10 and incubated at 37°C in a humidified atmosphere of 5% CO_2 for ~4 h. Afterward, the relative absorbance was measured at 450 nm using a fluorescence microplate reader, as previously described.[90,93–95]

Wound Healing/Cell Migration Assay

For the wound-healing assay, ~90% confluent cells in 60 mm culture dishes were treated with mitomycin C (MMC; 10 μg/mL) for 3 h, after which, the cells were scratched with a 200-μL pipette tip. The indicated wound areas in the dishes were marked, and photos were taken every 12 h. The pictures were analyzed in ImageJ, and the wound closure percentage (%) was determined, as previously described.[90]

Sphere Formation Assay

The indicated cells (1 × 10^5 cells) were seeded onto noncoated Petri dishes with sphere-forming medium containing serum-free DMEM/F12 media with B27 supplement, 20 ng/mL EGF (Sigma-Aldrich), 10 μg/mL insulin (Sigma-Aldrich), and 1% bovine serum albumin (Sigma-Aldrich). After 6 days, colonies were gently collected into conical tubes (SPL Lifesciences, Pocheon, South Korea) and stained with crystal violet (Sigma-Aldrich). Finally, the colonies were disassociated using 0.25% trypsin-EDTA (1×; Gibco, Thermo Fisher Scientific, Waltham, MA, USA), after which, the disassociated cells were counted and presented as the percent (%) of sphere-forming cells, as previously described.[90,94]

Drug-Resistance Assay

For drug-resistance analysis, 1 × 10^5 cells were seeded onto 12-well dishes and incubated overnight at 5% CO_2 and 37°C. The cells were then treated with 0.5 μM doxorubicin and incubated for another

48 h at 5% CO_2 and 37°C. After 48 h of incubation, the cells were counted and presented as the percent (%) of surviving cells, as previously described.[90]

Luciferase Reporter Assay

For the luciferase assay, the indicated cells (1×10^5 cells/well) were seeded onto 12-well plates and transiently transfected with 1 μg of either *HES1* or *HES5* luciferase plasmid using HyliMax transfection reagent (1:3 ratio; Dojindo).[96] The cells were harvested after 48 h post-transfection, and luciferase activity was measured using a luminometer (Veritas microplate luminometer; Turnor Biosystems, Sunnyvale, CA, USA). Luciferase activity was normalized to β-galactosidase expression levels.

ADAM17 Activity Assay

ADAM17 activity assay was performed using an ADAM17 Activity Assay Kit (CSB-E09315h; Cusabio Technology, Houston, TX, USA), according to the manufacturer's instructions.[97] Briefly, 1×10^7 cells were harvested in 1 mL of ice-cold PBS (pH 7.2–7.4) with protease inhibitor and stored at −20°C overnight. After two freeze-thaw cycles, the cell lysates were centrifuged for 5 min at 5,000 × *g* at 2°C–8°C, and then the supernatant was collected and stored at −20°C until future use, after measurement of the protein concentrations in the supernatant. Approximately 15 mg of protein was mixed with substrate for 15–30 min, and the optical density (OD) of each well was determined within 5 min using a microplate reader (x-Mark spectrophotometer; Bio-Rad Laboratories).

CoIP Assay

To analyze protein interactions, a coIP assay was performed with the indicated samples. Briefly, 400 μg of cell lysate was pretreated with 30 μL Protein A/G Agarose beads (Santa Cruz Biotechnology) to remove nonspecific IgG. The supernatant was then collected in new tubes and incubated overnight with 3–4 μg primary antibodies (anti-GPR50 or anti-ADAM17) and rabbit IgG on an agitator at 4°C. Subsequently, Protein A/G Agarose was added, and the mixture was incubated for an additional 3 h and spun down at 4,000 × *g* for 1 min. The pellets were then washed thrice with ice-cold cell lysis buffer, after which, the immunoprecipitated proteins were analyzed by western blotting, as described above.

Statistical Analysis

All experiments were performed three times, and the data are presented as the mean ± standard deviation (SD). For statistical analysis, an unpaired t test was performed between two groups (control versus treated), and p values <0.05 were considered statistically significant.

Data Availability

All data referenced in the manuscript can be downloaded from websites indicated in the Materials and Methods section.

SUPPLEMENTAL INFORMATION

Supplemental Information can be found online at https://doi.org/10.1016/j.omto.2020.04.002.

AUTHOR CONTRIBUTIONS

S.K.S. conceived of and participated in the study design, performed the experiments, analyzed the data, and wrote the manuscript. H.Y.C., G.-M.Y., P.K.B., K.K., G.-H.K., and M.G. performed some experiments and analyzed the data. S.-G.C. designed the study, reorganized the data, and wrote and edited the manuscript. All authors reviewed and approved the manuscript.

CONFLICTS OF INTEREST

The authors declare no competing interests.

ACKNOWLEDGMENTS

This study was funded by grants from the National Research Foundation (NRF) of the Korean government (NRF-2019M3A9H1030682 and NRF-2015R1A5A1009701).

REFERENCES

1. Bray, F., Ferlay, J., Soerjomataram, I., Siegel, R.L., Torre, L.A., and Jemal, A. (2018). Global cancer statistics 2018: GLOBOCAN estimates of incidence and mortality worldwide for 36 cancers in 185 countries. CA Cancer J. Clin. *68*, 394–424.
2. Kabbach, G., Assi, H.A., Bolotin, G., Schuster, M., Lee, H.J., and Tadros, M. (2015). Hepatobiliary Tumors: Update on Diagnosis and Management. J. Clin. Transl. Hepatol. *3*, 169–181.
3. Chu, K.K.W., and Cheung, T.T. (2015). Update in management of hepatocellular carcinoma in Eastern population. World J. Hepatol. *7*, 1562–1571.
4. Shao, Y.-Y., Shau, W.-Y., Chan, S.-Y., Lu, L.-C., Hsu, C.-H., and Cheng, A.-L. (2015). Treatment efficacy differences of sorafenib for advanced hepatocellular carcinoma: a meta-analysis of randomized clinical trials. Oncology *88*, 345–352.
5. Kitano, K., Murayama, T., Sakamoto, M., Nagayama, K., Ueno, K., Murakawa, T., and Nakajima, J. (2012). Outcome and survival analysis of pulmonary metastasectomy for hepatocellular carcinoma. Eur. J. Cardiothorac. Surg. *41*, 376–382.
6. Li, T., Fan, J., Qin, L.X., Zhou, J., Sun, H.C., Qiu, S.J., Ye, Q.H., Wang, L., and Tang, Z.Y. (2011). Risk factors, prognosis, and management of early and late intrahepatic recurrence after resection of primary clear cell carcinoma of the liver. Ann. Surg. Oncol. *18*, 1955–1963.
7. Rask-Andersen, M., Almén, M.S., and Schiöth, H.B. (2011). Trends in the exploitation of novel drug targets. Nat. Rev. Drug Discov. *10*, 579–590.
8. Spiegelberg, B.D., and Hamm, H.E. (2007). Roles of G-protein-coupled receptor signaling in cancer biology and gene transcription. Curr. Opin. Genet. Dev. *17*, 40–44.
9. Thomas, S.M., Bhola, N.E., Zhang, Q., Contrucci, S.C., Wentzel, A.L., Freilino, M.L., Gooding, W.E., Siegfried, J.M., Chan, D.C., and Grandis, J.R. (2006). Cross-talk between G protein-coupled receptor and epidermal growth factor receptor signaling pathways contributes to growth and invasion of head and neck squamous cell carcinoma. Cancer Res. *66*, 11831–11839.
10. Ahmad, R., Wojciech, S., and Jockers, R. (2015). Hunting for the function of orphan GPCRs - beyond the search for the endogenous ligand. Br. J. Pharmacol. *172*, 3212–3228.
11. Levoye, A., Dam, J., Ayoub, M.A., Guillaume, J.L., and Jockers, R. (2006). Do orphan G-protein-coupled receptors have ligand-independent functions? New insights from receptor heterodimers. EMBO Rep. *7*, 1094–1098.
12. Rezgaoui, M., Süsens, U., Ignatov, A., Gelderblom, M., Glassmeier, G., Franke, I., Urny, J., Imai, Y., Takahashi, R., and Schaller, H.C. (2006). The neuropeptide head activator is a high-affinity ligand for the orphan G-protein-coupled receptor GPR37. J. Cell Sci. *119*, 542–549.
13. Ito, J., Ito, M., Nambu, H., Fujikawa, T., Tanaka, K., Iwaasa, H., and Tokita, S. (2009). Anatomical and histological profiling of orphan G-protein-coupled receptor expression in gastrointestinal tract of C57BL/6J mice. Cell Tissue Res. *338*, 257–269.

14. Khalil, B.D., Hsueh, C., Cao, Y., Abi Saab, W.F., Wang, Y., Condeelis, J.S., Bresnick, A.R., and Backer, J.M. (2016). GPCR Signaling Mediates Tumor Metastasis via PI3Kβ. Cancer Res. *76*, 2944–2953.

15. Herr, D.R. (2012). Potential use of G protein-coupled receptor-blocking monoclonal antibodies as therapeutic agents for cancers. Int. Rev. Cell Mol. Biol. *297*, 45–81.

16. Dorsam, R.T., and Gutkind, J.S. (2007). G-protein-coupled receptors and cancer. Nat. Rev. Cancer *7*, 79–94.

17. Tang, X., Jin, R., Qu, G., Wang, X., Li, Z., Yuan, Z., Zhao, C., Siwko, S., Shi, T., Wang, P., et al. (2013). GPR116, an adhesion G-protein-coupled receptor, promotes breast cancer metastasis via the Gαq-p63RhoGEF-Rho GTPase pathway. Cancer Res. *73*, 6206–6218.

18. Feigin, M.E., Xue, B., Hammell, M.C., and Muthuswamy, S.K. (2014). G-protein-coupled receptor GPR161 is overexpressed in breast cancer and is a promoter of cell proliferation and invasion. Proc. Natl. Acad. Sci. USA *111*, 4191–4196.

19. Dufourny, L., Levasseur, A., Migaud, M., Callebaut, I., Pontarotti, P., Malpaux, B., and Monget, P. (2008). GPR50 is the mammalian ortholog of Mel1c: evidence of rapid evolution in mammals. BMC Evol. Biol. *8*, 105.

20. Jockers, R., Delagrange, P., Dubocovich, M.L., Markus, R.P., Renault, N., Tosini, G., Cecon, E., and Zlotos, D.P. (2016). Update on melatonin receptors: IUPHAR Review 20. Br. J. Pharmacol. *173*, 2702–2725.

21. Thomson, P.A., Wray, N.R., Thomson, A.M., Dunbar, D.R., Grassie, M.A., Condie, A., Walker, M.T., Smith, D.J., Pulford, D.J., Muir, W., et al. (2005). Sex-specific association between bipolar affective disorder in women and GPR50, an X-linked orphan G protein-coupled receptor. Mol. Psychiatry *10*, 470–478.

22. Bechtold, D.A., Sidibe, A., Saer, B.R.C., Li, J., Hand, L.E., Ivanova, E.A., Darras, V.M., Dam, J., Jockers, R., Luckman, S.M., and Loudon, A.S. (2012). A role for the melatonin-related receptor GPR50 in leptin signaling, adaptive thermogenesis, and torpor. Curr. Biol. *22*, 70–77.

23. Anyanwu, U.N. (2014). Characterising the role of GPR50 in neurodevelopment and lipid metabolism. PhD thesis (Edinburgh Medical School).

24. Grünewald, E., Kinnell, H.L., Porteous, D.J., and Thomson, P.A. (2009). GPR50 interacts with neuronal NOGO-A and affects neurite outgrowth. Mol. Cell. Neurosci. *42*, 363–371.

25. Khan, M.Z., He, L., and Zhuang, X. (2016). The emerging role of GPR50 receptor in brain. Biomed. Pharmacother. *78*, 121–128.

26. Choi, H.Y., Saha, S.K., Kim, K., Kim, S., Yang, G.-M., Kim, B., Kim, J.H., and Cho, S.G. (2015). G protein-coupled receptors in stem cell maintenance and somatic reprogramming to pluripotent or cancer stem cells. BMB Rep. *48*, 68–80.

27. Wojciech, S., Ahmad, R., Belaid-Choucair, Z., Journé, A.-S., Gallet, S., Dam, J., Daulat, A., Ndiaye-Lobry, D., Lahuna, O., Karamitri, A., et al. (2018). The orphan GPR50 receptor promotes constitutive TGFβ receptor signaling and protects against cancer development. Nat. Commun. *9*, 1216.

28. Ahmad, R., Wojciech, S., and Jockers, R. (2018). GPR50 in TGFβ signaling and breast cancer (Impact Journals).

29. Zhan, T., Rindtorff, N., and Boutros, M. (2017). Wnt signaling in cancer. Oncogene *36*, 1461–1473.

30. Venkatesh, V., Nataraj, R., Thangaraj, G.S., Karthikeyan, M., Gnanasekaran, A., Kaginelli, S.B., Kuppanna, G., Kallappa, C.G., and Basalingappa, K.M. (2018). Targeting Notch signalling pathway of cancer stem cells. Stem Cell Investig. *5*, 5.

31. Sari, I.N., Phi, L.T.H., Jun, N., Wijaya, Y.T., Lee, S., and Kwon, H.Y. (2018). Hedgehog Signaling in Cancer: A Prospective Therapeutic Target for Eradicating Cancer Stem Cells. Cells *7*, 208.

32. Hanna, A., and Shevde, L.A. (2016). Hedgehog signaling: modulation of cancer properies and tumor mircroenvironment. Mol. Cancer *15*, 24.

33. Park, J.H., Shin, J.E., and Park, H.W. (2018). The Role of Hippo Pathway in Cancer Stem Cell Biology. Mol. Cells *41*, 83–92.

34. Lu, J., Ye, X., Fan, F., Xia, L., Bhattacharya, R., Bellister, S., Tozzi, F., Sceusi, E., Zhou, Y., Tachibana, I., et al. (2013). Endothelial cells promote the colorectal cancer stem cell phenotype through a soluble form of Jagged-1. Cancer Cell *23*, 171–185.

35. Ladi, E., Nichols, J.T., Ge, W., Miyamoto, A., Yao, C., Yang, L.-T., Boulter, J., Sun, Y.E., Kintner, C., and Weinmaster, G. (2005). The divergent DSL ligand Dll3 does not activate Notch signaling but cell autonomously attenuates signaling induced by other DSL ligands. J. Cell Biol. *170*, 983–992.

36. Mullendore, M.E., Koorstra, J.B., Li, Y.M., Offerhaus, G.J., Fan, X., Henderson, C.M., Matsui, W., Eberhart, C.G., Maitra, A., and Feldmann, G. (2009). Ligand-dependent Notch signaling is involved in tumor initiation and tumor maintenance in pancreatic cancer. Clin. Cancer Res. *15*, 2291–2301.

37. Bozkulak, E.C., and Weinmaster, G. (2009). Selective use of ADAM10 and ADAM17 in activation of Notch1 signaling. Mol. Cell. Biol. *29*, 5679–5695.

38. Li, W., Wang, D., Sun, X., Zhang, Y., Wang, L., and Suo, J. (2019). ADAM17 promotes lymph node metastasis in gastric cancer via activation of the Notch and Wnt signaling pathways. Int. J. Mol. Med. *43*, 914–926.

39. Guo, Z., Jin, X., and Jia, H. (2013). Inhibition of ADAM-17 more effectively down-regulates the Notch pathway than that of γ-secretase in renal carcinoma. J. Exp. Clin. Cancer Res. *32*, 26.

40. Jing, P., Sa, N., Liu, X., Liu, X., and Xu, W. (2016). MicroR-140-5p suppresses tumor cell migration and invasion by targeting ADAM10-mediated Notch1 signaling pathway in hypopharyngeal squamous cell carcinoma. Exp. Mol. Pathol. *100*, 132–138.

41. Zolkiewska, A. (2008). ADAM proteases: ligand processing and modulation of the Notch pathway. Cell. Mol. Life Sci. *65*, 2056–2068.

42. Liu, W.-H., and Chang, L.-S. (2012). Suppression of Akt/Foxp3-mediated miR-183 expression blocks Sp1-mediated ADAM17 expression and TNFα-mediated NFκB activation in piceatannol-treated human leukemia U937 cells. Biochem. Pharmacol. *84*, 670–680.

43. Van Schaeybroeck, S., Kalimutho, M., Dunne, P.D., Carson, R., Allen, W., Jithesh, P.V., Redmond, K.L., Sasazuki, T., Shirasawa, S., Blayney, J., et al. (2014). ADAM17-dependent c-MET-STAT3 signaling mediates resistance to MEK inhibitors in KRAS mutant colorectal cancer. Cell Rep. *7*, 1940–1955.

44. Yamashita, Y., Hishinuma, M., and Shimada, M. (2009). Activation of PKA, p38 MAPK and ERK1/2 by gonadotropins in cumulus cells is critical for induction of EGF-like factor and TACE/ADAM17 gene expression during in vitro maturation of porcine COCs. J. Ovarian Res. *2*, 20.

45. Wang, J., Ma, L., Weng, W., Qiao, Y., Zhang, Y., He, J., Wang, H., Xiao, W., Li, L., Chu, Q., et al. (2013). Mutual interaction between YAP and CREB promotes tumorigenesis in liver cancer. Hepatology *58*, 1011–1020.

46. Mizui, Y., Yamazaki, K., Sagane, K., and Tanaka, I. (1999). cDNA cloning of mouse tumor necrosis factor-alpha converting enzyme (TACE) and partial analysis of its promoter. Gene *233*, 67–74.

47. Szalad, A., Katakowski, M., Zheng, X., Jiang, F., and Chopp, M. (2009). Transcription factor Sp1 induces ADAM17 and contributes to tumor cell invasiveness under hypoxia. J. Exp. Clin. Cancer Res. *28*, 129.

48. Artavanis-Tsakonas, S., and Muskavitch, M.A.T. (2010). Notch: the past, the present, and the future. Curr. Top. Dev. Biol. *92*, 1–29.

49. Tang, X., Zhao, Y.Y., Dewald, J., Curtis, J.M., and Brindley, D.N. (2016). Tetracyclines increase lipid phosphate phosphatase expression on plasma membranes and turnover of plasma lysophosphatidate. J. Lipid Res. *57*, 597–606.

50. Xing, F., Okuda, H., Watabe, M., Kobayashi, A., Pai, S.K., Liu, W., Pandey, P.R., Fukuda, K., Hirota, S., Sugai, T., et al. (2011). Hypoxia-induced Jagged2 promotes breast cancer metastasis and self-renewal of cancer stem-like cells. Oncogene *30*, 4075–4086.

51. Li, J., Hand, L.E., Meng, Q.J., Loudon, A.S.I., and Bechtold, D.A. (2011). GPR50 interacts with TIP60 to modulate glucocorticoid receptor signalling. PLoS ONE *6*, e23725.

52. Levoye, A., Dam, J., Ayoub, M.A., Guillaume, J.L., Couturier, C., Delagrange, P., and Jockers, R. (2006). The orphan GPR50 receptor specifically inhibits MT1 melatonin receptor function through heterodimerization. EMBO J. *25*, 3012–3023.

53. Kopan, R., and Ilagan, M.X.G. (2009). The canonical Notch signaling pathway: unfolding the activation mechanism. Cell *137*, 216–233.

54. Fortini, M.E. (2009). Notch signaling: the core pathway and its posttranslational regulation. Dev. Cell *16*, 633–647.

55. Bigas, A., and Espinosa, L. (2016). Notch Signaling in Cell–Cell Communication Pathways. Curr. Stem Cell Rep. *2*, 349–355.

56. Gan, R.H., Wei, H., Xie, J., Zheng, D.P., Luo, E.L., Huang, X.Y., Xie, J., Zhao, Y., Ding, L.C., Su, B.H., et al. (2018). Notch1 regulates tongue cancer cells proliferation, apoptosis and invasion. Cell Cycle *17*, 216–224.

57. Lai, X.X., Li, G., Lin, B., and Yang, H. (2018). Interference of Notch 1 inhibits the proliferation and invasion of breast cancer cells: Involvement of the β-catenin signaling pathway. Mol. Med. Rep. *17*, 2472–2478.

58. Aster, J.C., Pear, W.S., and Blacklow, S.C. (2017). The Varied Roles of Notch in Cancer. Annu. Rev. Pathol. *12*, 245–275.

59. Ntziachristos, P., Lim, J.S., Sage, J., and Aifantis, I. (2014). From fly wings to targeted cancer therapies: a centennial for notch signaling. Cancer Cell *25*, 318–334.

60. Nowell, C.S., and Radtke, F. (2017). Notch as a tumour suppressor. Nat. Rev. Cancer *17*, 145–159.

61. Lu, J., Xia, Y., Chen, K., Zheng, Y., Wang, J., Lu, W., Yin, Q., Wang, F., Zhou, Y., and Guo, C. (2016). Oncogenic role of the Notch pathway in primary liver cancer. Oncol. Lett. *12*, 3–10.

62. Villanueva, A., Alsinet, C., Yanger, K., Hoshida, Y., Zong, Y., Toffanin, S., Rodriguez-Carunchio, L., Solé, M., Thung, S., Stanger, B.Z., and Llovet, J.M. (2012). Notch signaling is activated in human hepatocellular carcinoma and induces tumor formation in mice. Gastroenterology *143*, 1660–1669.e7.

63. Seals, D.F., and Courtneidge, S.A. (2003). The ADAMs family of metalloproteases: multidomain proteins with multiple functions. Genes Dev. *17*, 7–30.

64. Edwards, D.R., Handsley, M.M., and Pennington, C.J. (2008). The ADAM metalloproteinases. Mol. Aspects Med. *29*, 258–289.

65. Rocks, N., Paulissen, G., El Hour, M., Quesada, F., Crahay, C., Gueders, M., Foidart, J.M., Noel, A., and Cataldo, D. (2008). Emerging roles of ADAM and ADAMTS metalloproteinases in cancer. Biochimie *90*, 369–379.

66. Mazzocca, A., Giannelli, G., and Antonaci, S. (2010). Involvement of ADAMs in tumorigenesis and progression of hepatocellular carcinoma: Is it merely fortuitous or a real pathogenic link? Biochim. Biophys. Acta *1806*, 74–81.

67. Le Gall, S.M., Bobé, P., Reiss, K., Horiuchi, K., Niu, X.D., Lundell, D., Gibb, D.R., Conrad, D., Saftig, P., and Blobel, C.P. (2009). ADAMs 10 and 17 represent differentially regulated components of a general shedding machinery for membrane proteins such as transforming growth factor alpha, L-selectin, and tumor necrosis factor alpha. Mol. Biol. Cell *20*, 1785–1794.

68. Lorenzen, I., Trad, A., and Grötzinger, J. (2011). Multimerisation of A disintegrin and metalloprotease protein-17 (ADAM17) is mediated by its EGF-like domain. Biochem. Biophys. Res. Commun. *415*, 330–336.

69. González-Foruria, I., Santulli, P., Chouzenoux, S., Carmona, F., Chapron, C., and Batteux, F. (2017). Dysregulation of the ADAM17/Notch signalling pathways in endometriosis: from oxidative stress to fibrosis. Mol. Hum. Reprod. *23*, 488–499.

70. van Tetering, G., van Diest, P., Verlaan, I., van der Wall, E., Kopan, R., and Vooijs, M. (2009). Metalloprotease ADAM10 is required for Notch1 site 2 cleavage. J. Biol. Chem. *284*, 31018–31027.

71. Inoue, A., Ishiguro, J., Kitamura, H., Arima, N., Okutani, M., Shuto, A., Higashiyama, S., Ohwada, T., Arai, H., Makide, K., and Aoki, J. (2012). TGFα shedding assay: an accurate and versatile method for detecting GPCR activation. Nat. Methods *9*, 1021–1029.

72. Sokolina, K., Kittanakom, S., Snider, J., Kotlyar, M., Maurice, P., Gandía, J., Benleulmi-Chaachoua, A., Tadagaki, K., Oishi, A., Wong, V., et al. (2017). Systematic protein-protein interaction mapping for clinically relevant human GPCRs. Mol. Syst. Biol. *13*, 918.

73. Höcker, M., Raychowdhury, R., Plath, T., Wu, H., O'Connor, D.T., Wiedenmann, B., Rosewicz, S., and Wang, T.C. (1998). Sp1 and CREB mediate gastrin-dependent regulation of chromogranin A promoter activity in gastric carcinoma cells. J. Biol. Chem. *273*, 34000–34007.

74. Li, L., He, S., Sun, J.-M., and Davie, J.R. (2004). Gene regulation by Sp1 and Sp3. Biochem. Cell Biol. *82*, 460–471.

75. Wang, L., Guan, X., Zhang, J., Jia, Z., Wei, D., Li, Q., Yao, J., and Xie, K. (2008). Targeted inhibition of Sp1-mediated transcription for antiangiogenic therapy of metastatic human gastric cancer in orthotopic nude mouse models. Int. J. Oncol. *33*, 161–167.

76. Wang, L.W., Li, Q., Hua, Z.L., Zhou, F., Keping, X., Daoyan, W., Yao, J., and Ajani, J. (2007). [Expression of transcription factor Sp1 in human gastric cancer tissue and its correlation with prognosis]. Zhonghua Zhong Liu Za Zhi *29*, 107–111.

77. Pore, N., Liu, S., Shu, H.K., Li, B., Haas-Kogan, D., Stokoe, D., Milanini-Mongiat, J., Pages, G., O'Rourke, D.M., Bernhard, E., and Maity, A. (2004). Sp1 is involved in Akt-mediated induction of VEGF expression through an HIF-1-independent mechanism. Mol. Biol. Cell *15*, 4841–4853.

78. Chuang, C.-W., Pan, M.-R., Hou, M.-F., and Hung, W.-C. (2013). Cyclooxygenase-2 up-regulates CCR7 expression via AKT-mediated phosphorylation and activation of Sp1 in breast cancer cells. J. Cell. Physiol. *228*, 341–348.

79. Chang, K.-W., Huang, Y.-L., Wong, Z.-R., Su, P.-H., Huang, B.-M., Ju, T.-K., and Yang, H.Y. (2013). Fibroblast growth factor-2 up-regulates the expression of nestin through the Ras-Raf-ERK-Sp1 signaling axis in C6 glioma cells. Biochem. Biophys. Res. Commun. *434*, 854–860.

80. Hu, H., Han, T., Zhuo, M., Wu, L.-L., Yuan, C., Wu, L., Lei, W., Jiao, F., and Wang, L.-W. (2017). Elevated COX-2 Expression Promotes Angiogenesis Through EGFR/p38-MAPK/Sp1-Dependent Signalling in Pancreatic Cancer. Sci. Rep. *7*, 470.

81. Liu, S., Liu, H., Qin, R., Shu, Y., Liu, Z., Zhang, P., Duan, C., Hong, D., Yu, J., and Zou, L. (2017). The cellular senescence of leukemia-initiating cells from acute lymphoblastic leukemia is postponed by β-Arrestin1 binding with P300-Sp1 to regulate hTERT transcription. Cell Death Dis. *8*, e2756.

82. Rhodes, D.R., Yu, J., Shanker, K., Deshpande, N., Varambally, R., Ghosh, D., Barrette, T., Pandey, A., and Chinnaiyan, A.M. (2004). ONCOMINE: a cancer microarray database and integrated data-mining platform. Neoplasia *6*, 1–6.

83. Rhodes, D.R., Kalyana-Sundaram, S., Mahavisno, V., Varambally, R., Yu, J., Briggs, B.B., Barrette, T.R., Anstet, M.J., Kincead-Beal, C., Kulkarni, P., et al. (2007). Oncomine 3.0: genes, pathways, and networks in a collection of 18,000 cancer gene expression profiles. Neoplasia *9*, 166–180.

84. Clough, E., and Barrett, T. (2016). The Gene Expression Omnibus Database. Methods Mol. Biol. *1418*, 93–110.

85. Aguirre-Gamboa, R., Gomez-Rueda, H., Martínez-Ledesma, E., Martínez-Torteya, A., Chacolla-Huaringa, R., Rodriguez-Barrientos, A., Tamez-Peña, J.G., and Treviño, V. (2013). SurvExpress: an online biomarker validation tool and database for cancer gene expression data using survival analysis. PLoS ONE *8*, e74250.

86. Cerami, E., Gao, J., Dogrusoz, U., Gross, B.E., Sumer, S.O., Aksoy, B.A., Jacobsen, A., Byrne, C.J., Heuer, M.L., Larsson, E., et al. (2012). The cBio cancer genomics portal: an open platform for exploring multidimensional cancer genomics data. Cancer Discov. *2*, 401–404.

87. Gao, J., Aksoy, B.A., Dogrusoz, U., Dresdner, G., Gross, B., Sumer, S.O., Sun, Y., Jacobsen, A., Sinha, R., Larsson, E., et al. (2013). Integrative analysis of complex cancer genomics and clinical profiles using the cBioPortal. Sci. Signal. *6*, pl1.

88. Liu, C., Xu, P., Lamouille, S., Xu, J., and Derynck, R. (2009). TACE-mediated ectodomain shedding of the type I TGF-β receptor downregulates TGF-β signaling. Mol. Cell *35*, 26–36.

89. Li, M.J., Husic, N., Lin, Y., and Snider, B.J. (2012). Production of Lentiviral Vectors for Transducing Cells from the Central Nervous System. J. Vis. Exp. (63), e4031 (Jove-J Vis Exp).

90. Saha, S.K., Choi, H.Y., Kim, B.W., Dayem, A.A., Yang, G.M., Kim, K.S., Yin, Y.F., and Cho, S.G. (2017). KRT19 directly interacts with β-catenin/RAC1 complex to regulate NUMB-dependent NOTCH signaling pathway and breast cancer properties. Oncogene *36*, 332–349.

91. Salmon, P., and Trono, D. (2007). Production and Titration of Lentiviral Vectors. Curr. Protoc. Human Genet. *Chapter 12*. Unit 12.10.

92. Nasri, M., Karimi, A., and Allahbakhshian Farsani, M. (2014). Production, purification and titration of a lentivirus-based vector for gene delivery purposes. Cytotechnology *66*, 1031–1038.

93. Saha, S.K., Kim, K., Yang, G.-M., Choi, H.Y., and Cho, S.-G. (2018). Cytokeratin 19 *(KRT19)* has a Role in the Reprogramming of Cancer Stem Cell-Like Cells to Less Aggressive and More Drug-Sensitive Cells. Int. J. Mol. Sci. *19*, 1423.

94. Saha, S.K., Yin, Y., Chae, H.S., and Cho, S.-G. (2019). Opposing Regulation of Cancer Properties via KRT19-Mediated Differential Modulation of Wnt/β-Catenin/Notch Signaling in Breast and Colon Cancers. Cancers (Basel) *11*, 99.

95. Saha, S.K., Yin, Y., Kim, K., Yang, G.-M., Dayem, A.A., Choi, H.Y., and Cho, S.G. (2017). Valproic Acid Induces Endocytosis-Mediated Doxorubicin Internalization and Shows Synergistic Cytotoxic Effects in Hepatocellular Carcinoma Cells. Int. J. Mol. Sci. *18*, 1048.

96. Lee, J.M., Kim, I.S., Kim, H., Lee, J.S., Kim, K., Yim, H.Y., Jeong, J., Kim, J.H., Kim, J.Y., Lee, H., et al. (2010). RORalpha attenuates Wnt/β-catenin signaling by PKCalpha-dependent phosphorylation in colon cancer. Mol. Cell *37*, 183–195.

97. Wang, Y.-W., Ren, H.-L., Wang, H.-F., Li, F.-D., Li, H.-H., and Zheng, Y.-H. (2015). Combining detection of Notch1 and tumor necrosis factor-α converting enzyme is a reliable biomarker for the diagnosis of abdominal aortic aneurysms. Life Sci. *127*, 39–45.

OMTO, Volume *17*

Supplemental Information

GPR50 Promotes Hepatocellular Carcinoma Progression via the Notch Signaling Pathway through Direct Interaction with ADAM17

Subbroto Kumar Saha, Hye Yeon Choi, Gwang-Mo Yang, Polash Kumar Biswas, Kyeongseok Kim, Geun-Ho Kang, Minchan Gil, and Ssang-Goo Cho

Supplemental Information:

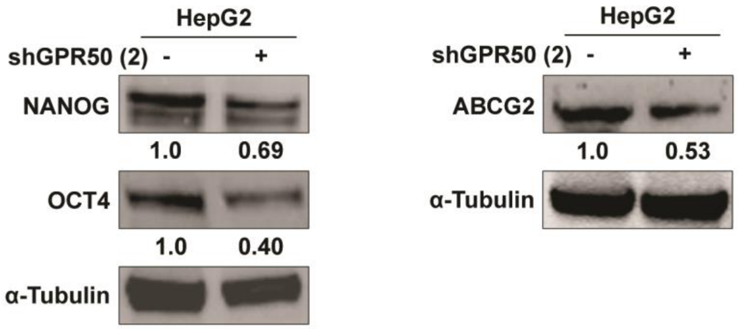

Supplementary Figure 1. Knockdown of *GPR50* led to suppressed protein expression of stemness and drug resistant markers in HCC. Protein expression of stemness markers (NANOG and OCT4) and drug resistant marker (ABCG2) were analyzed by western blot. α-Tubulin was used as an internal standard.

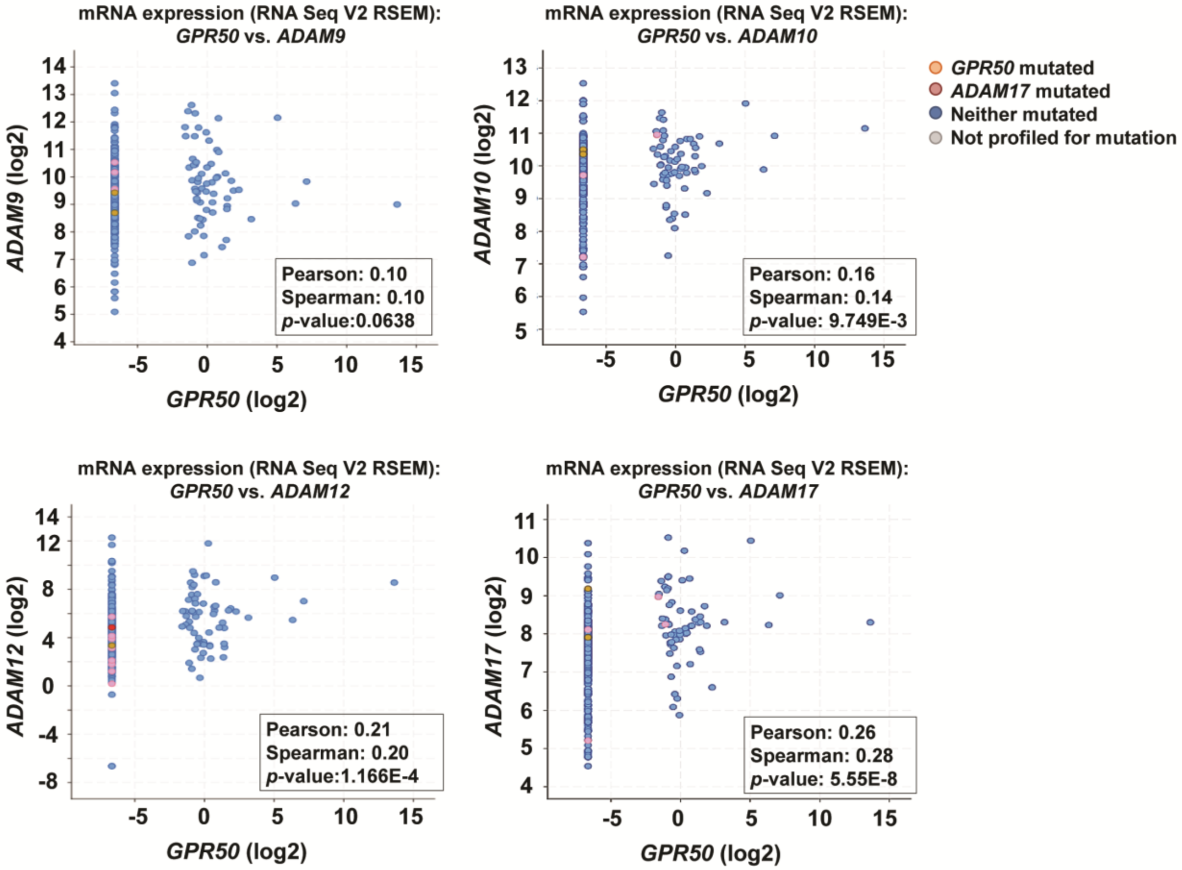

Supplementary Figure 2. GPR50 is positively co-expressed with ADAM family genes including *ADAM9*, *ADAM10*, *ADAM12*, and *ADAM17* in HCC. The co-expression data was plotted using TCGA data and downloaded from cBioPortal web.

Supplementary Table 1. *GPR50* is differentially expressed in various cancer types. Analysis of GEO datasets for *GPR50* mRNA expression in BRC, CEC, ESC, HCC, and LUC compared with normal breast, cervical, esophageal, liver, and lung tissue.

GEO/EMBL-EBI accession no.	Median expression (Normal sample)	Median expression (Cancer sample)	*p*-value
**Breast**			
GSE8977	-0.129 (15)	-0.459 (7)	0.019
EGAS00000000083	-0.14 (144)	-0.17 (14)	0.039
**Cervical**			
GSE7410	0.194 (5)	-0.131 (40)	1.75E-4
GSE9750	-2.66 (21)	-2.983 (32)	0.132
**Esophagus**			
GSE13898	0.225 (28)	0.224 (75)	7.29E-6
GSE23400	-1.199 (53)	-1.296 (53)	0.003
**Liver**			
GSE14520	3.601 (220)	3.524 (225)	<0.0001
GSE54236	4.19 (80)	4.323 (78)	0.475
**Lung**			
GSE31210	-4.885 (20)	-4.352 (226)	0.003
GSE2514	-1.724 (19)	-1.617 (20)	0.071

Supplementary Table 2. List of primers used for knockdown and overexpression of GPR50.

Accession no.	shRNA	Oligomers (5′–3′)	Target site
NM_004224.3	*GPR50*-(1)	TCGTGGGTTTCTGCTACGTGAG	747-767
	GPR50-(2)	GCTGACTCTGTCCATTTCAAG	1480-1500
	Scrambled	CCTAAGGTTAAGTCGCCCTCGCTC	Non-specific
**Plasmid name**	**Gene**	**Forward (5′–3′)**	**Reverse (5′–3′)**
pGEMR-T easy vector	*GPR50*	TATtctagaAGAGAGGGAGGCACGCTT (*Xba*I)	TATggatccTGCAGTAAGGCATCTCAC (*BamH*I)
pCDH-EF1-MCS-T2A-copGFP	*GPR50*		CGCggatccCACAGCCATTTCATCAGG (*BamH*I)

Underlined sequences represent the restriction sites.

Supplementary Table 3. Primer sequences used for qRT-PCR.

Accession no.	Gene	Froward Primer (5'→3')	Reverse Primer (5'→3')
NM_004224.3	*GPR50*	CCATCGTTGTAGACCTAATCG	CAGCATCAAAGGGTATGGGTA
NM_002046.5	*GAPDH*	AATCCCATCACCATCTTCCAG	CACGATACCAAAGTTGTCATGG
NM_001285986.1	*OCT4*	GTCCCAGGACATCAAAGCTC	CTCCAGGTTGCCTCTCACTC
NM_003106.3	*SOX2*	ACACCAATCCCATCCACACT	GCAAGAAGCCTCTCCTTGAA
NM_024865.3	*NANOG*	ATACCTCAGCCTCCAGCAGA	GCAGGACTGCAGAGATTCCT
NM_002467.4	*c-MYC*	CTCGGATTCTCTGCTCTC	TCGCCTCTTGACATTCTC
NM_001314052.1	*KLF4*	GAACTGACCAGGCACTACCG	TTCTGGCAGTGTGGGTCATA
NM_001348946.1	*P-GP*	GAGGAAGACATGACCAGGTA	CTGTCGCATTATAGCATGAA
NM_004827.2	*ABCG2*	TTATCCGTGGTGTGTCTGGAG	TCCTGCTTGGAAGGCTCTATG
NM_004996.3	*ABCC1*	GCCGGTGAAGGTTGTGTACT	CTGACGAAGCAGATGTGGAA
NM_001163993.2	*ABCB5*	GAGAGACAGTCGCCTTGGTC	CCACGATTGTAGTCCGACCT
NM_000689.4	*ALDH1A1*	CTGCTGGCGACAATGGAGT	GTCAGCCCAACCTGCACAG
NM_000214.3	*JAG1*	CGGGATTTGGTTAATGGTTATC	ATAGTCACTGGCACGGTTGTAGCAC
NM_002226.5	*JAG2*	GGTCGTACTTGCACTCACAATACC	GTAGCAAGGCAGAGGGTTGC
NM_005618.4	*DLL1*	CCAAGCCCTGCAAGAATGGA	GGTGGGCAGGTACAGGAGTA
NM_016941.3	*DLL3*	GAGACACCCAGGTCCTTTGA	CAGTGGCAGATGTAGGCAGA
NM_019074.4	*DLL4*	GTGGGTCAGAACTGGTTATGGA	TGCAGATGACCCGGTAAGAGT
NM_017617.3	*NOTCH1*	GACGGACCCAACACTTACAC	TCAGGCAGAAGCAGAGGTAG
NM_014757.4	*MAML1*	CACCAGCCACCGAGTAACTT	AACAGGGAGTTCTGCTCGTG
NM_005349.3	*RBPjK*	GAACAAATGGAACGCGATGG	GATGACTTTTATCCGCTTGCTG
NM_005524.3	*HES1*	GGCTAAGGTGTTTGGAGGCT	GGTGGGTTGGGGAGTTTAGG
NM_001130145.3	*YAP1*	TGAACAAACGTCCAGCAAGATAC	CAGCCCCCAAAATGAACAGTAG
NM_012242.4	*DKK1*	GGGCGGGAATAAGTACCAG	CATAGCGTGACGCATGCAG
NM_005269.3	*GLI1*	TTCCTACCAGAGTCCCAAGT	CCCTATGTGAAGCCCTATTT
NM_003816.3	*ADAM9*	GGTGCTGGTGATGTGCTG	CTGCGTGGCTCCTTGAAC
NM_001110.4	*ADAM10*	ATGGATTGTGGCTCATTGGT	TGCCTGGAAGTGGTTTAGGA
NM_001288975.1	*ADAM12*	CAGGAAGGACTTGGAGAC	AGCAGCGATTCATACATTC
NM_003183.6	*ADAM17*	GTATCTGAACAACGACACCTG	CCTCCTGGCACTTCTTCTG
NM_138473.3	*SP1*	TCCAGACCATTAACCTCAGTGC	TGTATTCCATCACCACCAGCC
NM_003110.6	*SP2*	CCAGCCTACCCCAAGGAAAC	GGGAGCCCTGAATCTGAAGTAT
NM_001172712.1	*SP3*	GCTTGCACCTGTCCCAACTGTA	CTCCAGAATGCCAACGCAGA
NM_003112.5	*SP4*	ATGGCTACAGAAGGAGGGAAAAC	TTGACCAGGGGTGGAAGAATTAC
